# Strategies for enhancing cancer chemodynamic therapy performance

**DOI:** 10.1002/EXP.20210238

**Published:** 2022-03-07

**Authors:** Deblin Jana, Yanli Zhao

**Affiliations:** ^1^ Division of Chemistry and Biological Chemistry School of Physical and Mathematical Sciences Nanyang Technological University Singapore Singapore

**Keywords:** chemodynamic therapies, Fenton/Fenton‐like reactions, nanomaterials, reactive oxygen species, stimuli

## Abstract

Chemodynamic therapy (CDT) has emerged to be a frontrunner amongst reactive oxygen species‐based cancer treatment modalities. CDT utilizes endogenous H_2_O_2_ in tumor microenvironment (TME) to produce cytotoxic hydroxyl radicals (•OH) via Fenton or Fenton‐like reactions. While possessing advantages such as tumor specificity, no need of external stimuli, and low side effects, practical applications of CDT are still impeded owing to the heterogeneity, complexity, and reductive environment of TME. Over the past couple of years, strategies to enhance CDT for efficient tumor regression are in rapid development in synergy with the growth of nanomedicine. In this review, we initially outline the fundamental understanding of Fenton and Fenton‐like reactions and their relationship with CDT. Subsequently, the development in the design of nanosystems for CDT is highlighted in a general manner. Furthermore, recent advancement of the strategies to augment Fenton reactions in TME for enhanced CDT is discussed in detail. Finally, perspectives toward the future development of CDT for better therapeutic outcome are presented. This review is expected to draw attention for collaborative research on CDT in the best interest of its future clinical applications.

## Graphical abstract

Chemodynamic therapy (CDT) utilizes endogenous H_2_O_2_ in tumor microenvironment to produce cytotoxic hydroxyl radicals through Fenton or Fenton‐like reactions. This review outlines the fundamental understanding of Fenton and Fenton‐like reactions and their relationship with CDT and highlights recent research advancements in the design of nanosystems to augment Fenton or Fenton‐like reactions for enhanced CDT. Perspectives toward the future development of CDT strategies for higher therapeutic outcome are also discussed.

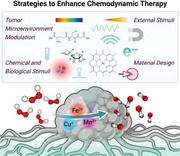

## 1 INTRODUCTION

Cancer mortality cases follow a rising curve owing to the low prognosis and rapid metastasis.^[^ ^1^ ^]^ Current clinical treatment methods of cancer include chemotherapy, surgery, and radiotherapy.^[^ ^2^ ^]^ However, complete eradication of tumor and nullifying the possibility of future metastasis through these methods are still difficult to achieve. Additionally, non‐specificity and drug resistance of chemotherapeutic drugs, as well as post‐surgery trauma from surgical removal of tumor and unavoidable side effects by radiotherapy limit efficient applications of current cancer treatment methodologies.^[^ ^3^ ^]^ Urgent need of minimally invasive treatment strategies has compelled researchers to investigate alternative approaches.^[^ ^4^ ^]^

Reactive oxygen species (ROS) in cancer cells are overexpressed as compared to that of the normal cells, thereby making cancer cells more prone to oxidative damages.^[^ ^5^ ^]^ Even though current chemotherapy and radiotherapy modalities utilize ROS generation pathways for cancer treatment, there is still an urge of finding new modalities for ROS‐based therapy by utilizing tumor associated nutrients. Tumor microenvironment (TME) consists of several defining characteristics, that is, hypoxia, overexpression of hydrogen peroxide (H_2_O_2_) and glutathione (GSH), low pH, aberrant vessels, and elevated consumption of cell nutrients.^[^ ^6^ ^]^ ROS related mechanisms such as photodynamic therapy (PDT), sonodynamic therapy (SDT), and enhanced radiotherapy have been in development based on the sensitive response activity of TME.^[^ ^7^ ^]^ These strategies generally use external stimuli of light, ultrasound, or X‐ray for the activation of nanoagents to amplify tumor oxidative stress. Utilization of such noninvasive or minimally invasive paradigms is at the frontier of the cancer therapy research. If the characteristics of TME are considered in synergy of ROS generation approach, more efficient solutions toward tumor ablation would be achieved.^[^ ^8^ ^]^

In 2016, Shi and coworkers reported chemodynamic therapy (CDT) by achieving the synergy of TME and Fenton reaction to generate tumor‐specific and cytotoxic hydroxyl radical (•OH) in cancer cells.^[^ ^9^ ^]^ CDT utilizes Fenton or Fenton‐like reactions to convert endogenous H_2_O_2_ to highly pernicious •OH, which in turn can induce tumor apoptosis by protein inactivation, phospholipid peroxidation, and DNA damage.^[^ ^10^ ^]^ CDT is reliant on the overexpression of H_2_O_2_ and mild acidity of TME, and highly specific toward cancerous tissues, while showing less or no toxicity to healthy cells. Several other factors like high level of catalytic ROS generation, less reliance on external stimuli, deep tissue treatment ability, and non‐multidrug resistance make CDT highly promising over other ROS‐based therapy methods. By avoiding the usage of O_2_ in the catalytic process, CDT demonstrates a new paradigm of hypoxic tumor treatment.^[^ ^11^ ^]^ When combined with stimuli to augment the Fenton or Fenton‐like reactions, the efficacy and sensitivity of CDT could be enhanced manyfold.^[^ ^12^, ^13^ ^]^

Fenton chemistry is generally employed in water treatment applications and has garnered ample attention for research.^[^ ^14^ ^]^ The development in the materials design and theoretical optimization has considerably enhanced Fenton reaction performance in recent years, and thereby such studies act as a guide toward enhancing the efficacy of CDT. Primarily being a catalytic reaction, Fenton chemistry is often amplified by external stimuli such as heat,^[^ ^10^ ^]^ light,^[^ ^15^ ^]^ and ultrasound.^[^ ^16^ ^]^ the regression of tumor cell antioxidant mechanism and modulation of TME to favor Fenton or Fenton‐like reactions can noticeably augment •OH generation. A range of chemodynamic agents, for example, Fe^2+^,^[^ ^17^ ^]^ Cu^+^,^[^ ^18^ ^]^ Mn^2+^,^[^ ^19^ ^]^ Mo^4+^,^[^ ^20^ ^]^ W^4+^,^[^ ^21^ ^]^ and Ti^3+^‐based nanomaterials,^[^ ^22^ ^]^ have been designed with enhanced CDT efficacy over the years. On the other hand, the inability to eradicate tumor entirely by single therapeutic approach of CDT paved the path of research toward newer system design for multimodal therapy and enhanced CDT.

**SCHEME 1:**
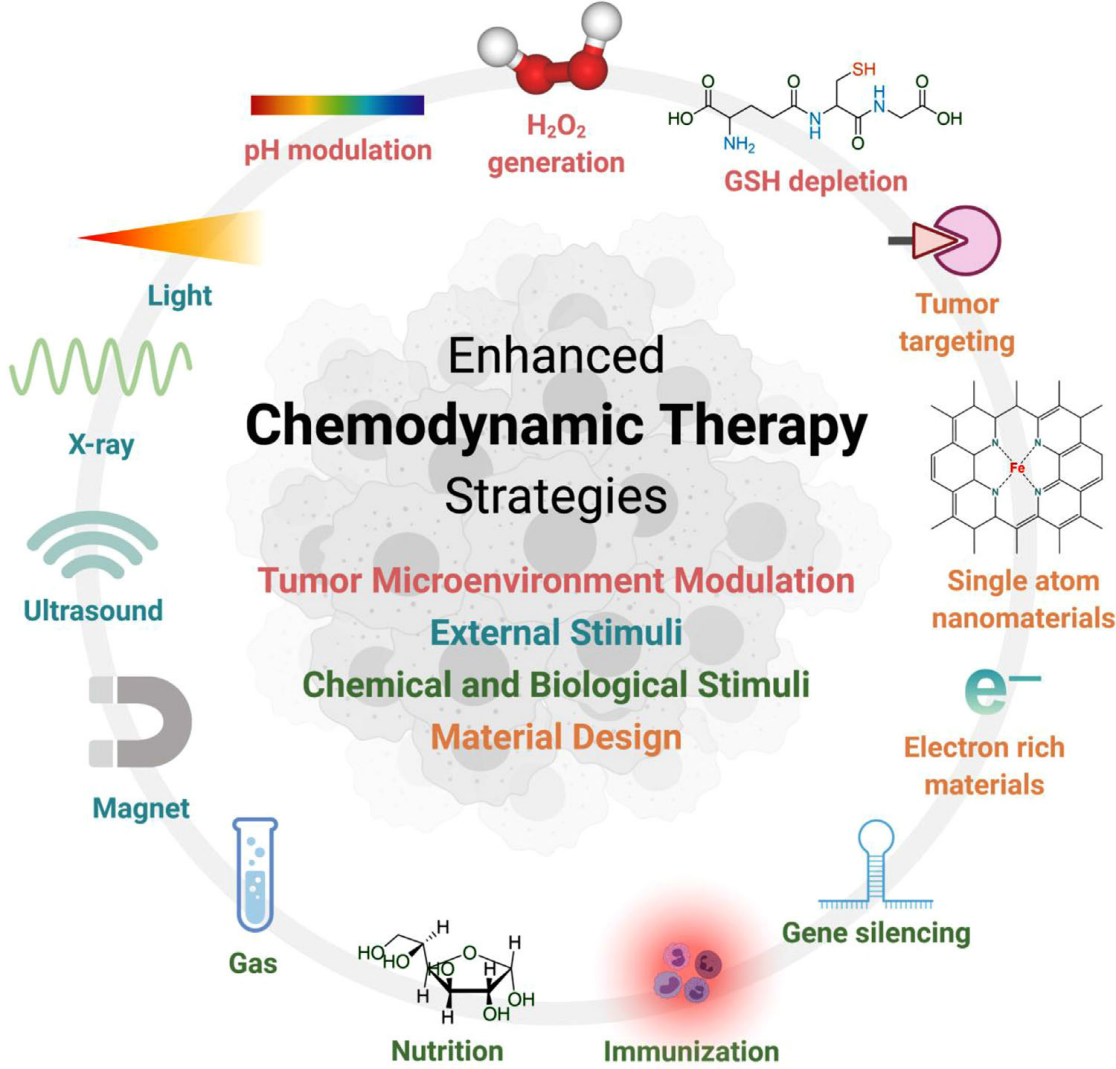
Overview of the strategies to enhance CDT through Fenton chemistry. Created with permission from BioRender.com

In this review, we focus on recent significant advancements in the structural designs of chemodynamic agents and the strategies to enhance the CDT performance in tumor for amplified therapeutic effect (Scheme 1). At the beginning, we discuss the basic information on Fenton/Fenton‐like reaction, followed by a brief summary on different types of CDT nanosystems, including metal‐based nanosystems, framework structures, and carbon‐based nanosystems. Furthermore, the strategies to optimize the performance of Fenton reaction and CDT are emphasized. The strategies are focused on the modulation of TME, the utilization of external stimuli, the nanoparticle designs to incorporate chemical and biological stimuli, and others, aiming toward better understanding of the design principles of CDT agents for best possible therapeutic outcome. Finally, the main hurdles for the clinical applications of nanosystems for CDT are reviewed thoroughly, along with brief perspectives to tackle such challenges.

## 2 FENTON REACTION IN CHEMODYNAMIC THERAPY

From the moment Fenton reaction was reported, it has been extensively used for water treatment research. Broadly, the reaction between Fe^2+^ and H_2_O_2_ could produce •OH, which in turn could degrade the pollutants of water.^[^ ^14^ ^]^ Several factors like high demand of H_2_O_2_ and maintaining narrow optimum pH window are important for suitable industrial performance of Fenton reaction. Researchers have noted that the Fenton reaction can impart oxidative damage toward the nutrients (such as, DNA, protein, or lipid) of cancer cells, leading to therapy. Overexpression of H_2_O_2_ (∼100 μM) and mild acidity of TME create proper setting for Fenton reaction (Fe^2+^ + H_2_O_2_ → Fe^3+^ + •OH + OH^−^) to proceed.^[^ ^23^ ^]^ Given the very short half‐life of •OH (10^−9^ s), it is rational to design Fe‐based nanosystems for targeted intracellular Fenton reaction with noninvasive treatment efficacy.^[^ ^24^ ^]^ To further alleviate the narrow window of acidic pH for efficient cancer CDT, several nanosystems and nanozymes have been designed by utilizing other transition metal ions, that is, Mo^4+^,^[^ ^20^ ^]^ Ti^3+^,^[^ ^22^ ^]^ Cu^+^,^[^ ^25^ ^]^ Mn^2+^,^[^ ^26^ ^]^ Ag^+^,^[^ ^27^ ^]^ and V^2+^.^[^ ^28^ ^]^ For example, Fenton‐like reactions by Cu^+^ can proceed ∼ 160 folds faster than that of Fe^2+^ and reported to be more proficient in generating noxious •OH in TME (pH 6.5–6.9).^[^ ^29^ ^]^ Additionally, the byproduct of such Fenton‐like reaction, Cu^2+^, can be reduced back to Cu^+^ ion by overexpressed endogenous GSH. Intracellular redox hemostasis is maintained by GSH and thus a depletion of the same can alleviate antioxidant barrier for enhanced Fenton or Fenton‐like reactions. Such transition metal induced Fenton‐like reactions offer several advantages, such as optimal performance in near‐neutral conditions and high natural abundance of structurally different oxide compounds.^[^ ^30^ ^]^ Although Fe‐based nanocatalyts require low pH conditions and high catalyst doses, they possess the optimum activities with minimal H_2_O_2_ concentration, and low energy of activation compared to other species.^[^ ^31^ ^]^ Active redox cycle feasibility in the pH condition, catalyst loading, and stability of oxidation products are some factors to be considered before precise design of Fenton/Fenton‐like reaction based nanomedicine. The complex nature of TME as well as the complexity of preparing an “all‐in‐one” chemodynamic agent often hinder the full potential of chemodynamic cancer therapy. Thereby, the design of suitable Fenton nanosystems and the modulation of TME in favor of CDT are of foremost concern for this research direction.

## 3 DIFFERENT KINDS OF NANOSYSTEMS

Owing to the irreplaceable role of the catalysts in Fenton reactions, the selection of a suitable nanoplatform is of utmost importance. Structure‐activity relationship of different nanosystems with CDT is a comprehensive research direction. In the following section, we discuss the recent development of a range of nanosystems, including metal‐based nanosystems, framework structures, and carbon‐based nanosystems, for CDT applications. Under metal‐based nanosystems, we cover metal oxides, metal chalcogenides, and metallic nanoparticles. Additionally, another subsection of framework structures and carbon‐based nanosystems is provided by incorporating assembled nanoparticles from organic small molecules and metal ions, metal‐organic frameworks (MOFs), and carbon‐based nanosystems. In this generalized approach, we overview different nanosystems comprising of Fenton‐active elements responsible for CDT.

### 3.1 Metal‐based nanosystems

**FIGURE 1:**
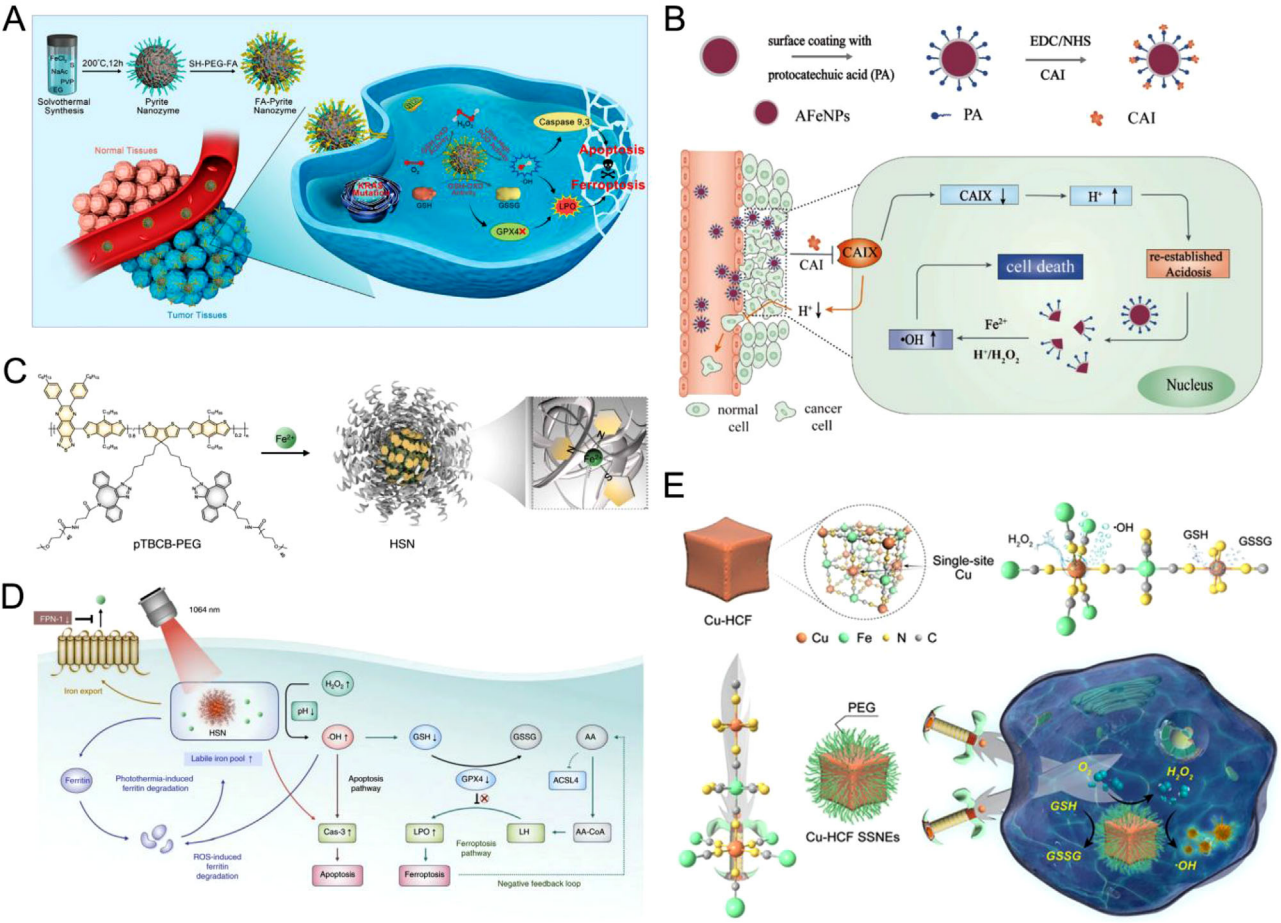
(A) Schematic illustration of the self‐cascade pyrite nanozyme with POD‐like catalytic activity and intrinsic GSH‐OXD mimicking ability for apoptosis−ferroptosis synergistic tumor therapy. Reproduced with permission.^[^ ^34^ ^]^ Copyright 2021, American Chemical Society. (B) Preparation process of AFeNPs@CAI and the schematic diagram of self‐enhanced CDT by AFeNPs@CAI. Reproduced with permission.^[^ ^35^ ^]^ Copyright 2019, Wiley‐VCH. (C) Chemical structure of pTBCB‐PEG, preparation of HSN, and proposed mechanism of iron chelation. (D) Proposed molecular mechanisms of HSN‐mediated NIR‐II photothermal ferrotherapy. Reproduced under the terms of the Creative Commons CC BY license.^[^ ^37^ ^]^ Copyright 2020, The Authors, published by Springer Nature. (E) Construction of Cu‐HCF SSNEs for tumor‐specific cascade enzymatic therapy. Reproduced with permission.^[^ ^40^ ^]^ Copyright 2021, Wiley‐VCH

As Fenton/Fenton‐like reactions rely mostly on the participating metal entity, metal‐based nanosystems are promising for CDT. Briefly, metal‐based nanosystems can be broadly categorized to metal oxides, metal chalcogenides, and metallic nanoparticles. For instance, Zhu et al. synthesized polyethylene glycol (PEG) coated cuprous oxide nanocrystals (Cu_2_O‐PEG) followed by doxorubicin (DOX) loading (DOX@Cu_2_O‐PEG) for DOX mediated chemotherapy and Cu^+^‐mediated CDT.^[^ ^32^ ^]^ Similarly, Yang and coworkers loaded ultrasmall γ‐Fe_2_O_3_ nanoparticles and glucose oxidase (GOx) into dendritic mesoporous silica (DMSN) spheres, synthesizing magnetic targeting nanoplatform (γ‐Fe_2_O_3_‐GOx‐DMSN).^[^ ^33^ ^]^ In acidic TME, the γ‐Fe_2_O_3_ nanoparticles could generate •OH from H_2_O_2_ that was produced as a side product of glucose consumption by GOx, through Fenton reaction. Like metal oxide nanoparticles, semiconductor‐like metal chalcogenide nanoparticles have been in use for CDT. Semiconductor nanoparticles are actively explored owing to their distinctive electrical and optical properties, and their incremental use in biological and clinical research is fascinating. Recently, Fan et al. synthesized a pyrite (FeS_2_) nanozyme with glutathione oxidase (GSHOx)‐ and peroxidase (POD)‐like activities for the depletion of GSH and abundant generation of •OH toward cancer therapy (Figure 1A).^[^ ^34^ ^]^ Additionally, electron deficient copper chalcogenides (e.g., Cu_2‐_ *_x_* S and Cu_2‐_ *_x_* Se) have been of high interest of studies for their plasmonic properties and Cu(I)‐mediated Fenton‐like reactions.

Moreover, metal‐based nanoclusters and noble metal‐based nanosystems are highly useful for CDT application due to their TME responsive activation and action. For example, Fe^0^ nanoparticles tend to release Fenton active Fe^2+^ ion more easily as compared to iron oxide nanoparticles. Shi and coworkers synthesized amorphous Fe^0^ nanocrystals (AFeNPs), which released ferrous ion in mildly acidic TME for CDT. To bridge the gap between mildly acidic TME and pH required for ideal Fenton reaction (pH 3–4), Bu and coworkers fabricated carbonic anhydrase IX inhibitor (CAI) on the surface of AFeNPs (AFeNPs@CAI) to reconfigure tumor acidosis (Figure 1B).^[^ ^35^ ^]^ The inhibition of overexpressed carbonic anhydrase IX in cancer cells resulted in extracellular alkalinity increase and intracellular acidity decrease, which in turn was beneficial for augmenting the efficiency of Fenton reaction and hence •OH‐mediated oxidative damage of tumor through self‐enhanced CDT. In addition, noble metal (e.g., Au, Pt, and Pd)‐based monometallic or multimetallic nanozymes have garnered much attention owing to their enzyme mimetic activities and have been employed in biosensing, antibacterial, and tumor treatment. Recently, we synthesized an ultrasmall trimetallic (Pd, Cu, and Fe) alloy nanozyme (PCF‐a), possessing synergistic glutathione peroxidase (GSH‐Px) and POD mimicking activities in TME.^[^ ^36^ ^]^ PCF‐a exhibited both photothermal and ultrasound augmented •OH generation from endogenous H_2_O_2_ for cancer therapy.

### 3.2 Framework structures and carbon‐based nanosystems

Apart from using inorganic nanosystems, polymers and amino acids can also be utilized as the backbone of the nanostructures. Such organic nanostructures are preferable owing to the structural uniformity, physiological stability, and biocompatibility, compared to the other nanomaterials. Zhang et al. fabricated a hybrid semiconducting nanozyme (HSN) through Fe^2+^‐chelation to amphiphilic semiconducting polymer PEGylated poly[(thiadiazoloquinoxaline‐alt‐benzodithiophene)‐ran‐(cyclopentadithiophene‐alt‐benzodithiophene)] (pTBCB‐PEG) (Figure 1C,D).^[^ ^37^ ^]^ HSN acted as an NIR‐II photothermal transducer, which in turn potentiated Fe^2+^‐mediated Fenton reaction to improve the outcome of therapy. Similarly, Li and coworkers synthesized self‐assembled copper–amino acid mercaptide nanoparticles (Cu‐Cys) for in situ GSH‐activation and subsequent H_2_O_2_‐reinforced CDT.^[^ ^29^ ^]^

MOFs have been of ample interests in cancer therapy due to their porosity and easily modifiable structural composition along with enzyme‐like properties in some cases.^[^ ^38^ ^]^ Well‐designed structural changes by utilizing Fenton‐active metal nodes can provide the multifunctionality for practical applications. Based on such characteristics, Tang et al. synthesized a dihydroartemisinin (DHA) loaded and CaCO_3_ coated Fe‐TCPP [(4,4,4,4‐(porphine‐5,10,15,20‐tetrayl) tetrakis(benzoic acid)] nMOF (nanoscale MOF) for multimodal therapy.^[^ ^39^ ^]^ In acidic and reductive TME, DHA is released and TCPP is activated, facilitating the synergistic Fe^2+^‐DHA‐mediated CDT, TCPP‐mediated PDT, and Ca^2+^‐DHA‐mediated oncosis therapy. Similarly, we developed a Prussian blue analogue‐based MOF, namely PEG‐modified copper hexacyanoferrate (Cu‐HCF), having GSHOx‐ and POD‐mimicking activities (Figure 1E).^[^ ^40^ ^]^ Cu‐HCF single‐site nanozymes (SSNEs) could deplete intracellular GSH and promote the conversion of single‐site Cu^2+^ species into Cu^+^ for augmented •OH generation through a Fenton‐type Haber–Weiss reaction, showing the elimination of cancer cells in vivo.

At the same time, carbon‐based nanomaterials are of interest because of their POD mimicking enzymatic activity to propel the decomposition of H_2_O_2_ into •OH. Zhang et al. fabricated GOx‐decorated N‐doped carbon (NC) nanoparticles as a biomimetic nanozyme (NC@GOx).^[^ ^41^ ^]^ GOx mediated starvation therapy could in turn reduce the expression of heat shock proteins, enhancing NIR‐laser mediated photothermal therapy (PTT). H_2_O_2_, generated as the byproduct of starvation therapy, could further augment CDT by NC@GOx for efficient tumor therapy. Additionally, various MXene nanocomposites, displaying good biocompatibility and photothermal properties, have been studied for tumor therapy applications. Wu et al. doped Fe^2+^ ion into the layers of Ti_3_C_2_ nanosheets, giving rise to a multifunctional nanoshell of Fe(II)‐Ti_3_C_2_ (FTC).^[^ ^42^ ^]^ FTC could show high photothermal conversion efficiency as well as Fenton reaction‐mediated CDT by Fe^2+^ ion.

## 4 STRATEGIES TO ENHANCE CHEMODYNAMIC THERAPY

TME is recognized for several distinctive characteristics like low pH, hypoxia, and overexpression of H_2_O_2_ and GSH.^[^ ^8^ ^]^ The focus of enhanced Fenton/Fenton‐like reaction in tumor is to enhance the generation of ROS by diminishing the possible blocking pathways. Such strategies can be roughly outlined into TME modulation, usage of external stimuli, utilization of chemical and biological stimuli, and design of the nanoplatforms (Table 1).^[^ ^16^, ^17^, ^22^, ^34^, ^35^, ^36^, ^40^, ^43^, ^44^, ^45^, ^46^, ^47^, ^48^, ^49^, ^50^, ^51^, ^52^, ^53^, ^54^, ^55^, ^56^, ^57^, ^58^, ^59^, ^60^, ^61^, ^62^, ^63^, ^64^, ^65^, ^66^, ^67^, ^68^, ^69^, ^70^, ^71^, ^72^, ^73^, ^74^, ^75^, ^76^, ^77^, ^78^, ^79^, ^80^, ^81^, ^82^, ^83^, ^84^, ^85^, ^86^, ^87^, ^88^, ^89^, ^90^, ^91^, ^92^, ^93^, ^94^, ^95^, ^96^, ^97^, ^98^, ^99^, ^100^, ^101^, ^102^, ^103^, ^104^, ^105^ ^]^

**TABLE 1:** Strategies to enhance CDT

**Strategies**	**Nanoplatform**	**Therapy mode**	**Cancer**	**Ref**.
*TME modulation*				
pH modulation	AFeNPs@CAI	CDT	MDA‐MB‐231	^[^ ^35^ ^]^
	FePt@FeOx@TAM‐PEG	CDT	4T1	^[^ ^43^ ^]^
	UCNP@PVP@MIL88B	CDT	U87MG	^[^ ^44^ ^]^
H_2_O_2_ generation	PTCG (EGCG, Pt‐OH, PEG‐b‐PPOH)	CDT/Chemotherapy	HepG2	^[^ ^17^ ^]^
	Copper hexacyanoferrate	CDT	4T1	^[^ ^40^ ^]^
	Den‐DOX‐Fe^3+^‐TA	CDT/Chemotherapy	U14	^[^ ^45^ ^]^
	PGC‐DOX (PEG‐GOx, CaCuP, DOX)	CDT/Chemotherapy	4T1	^[^ ^46^ ^]^
	ACC@DOX•Fe^2+^‐CaSi‐PAMAM‐FA/mPEG	CDT/Chemotherapy	A375, 4T1	^[^ ^47^ ^]^
	(MSNs@CaO_2_‐ICG)@LA	CDT/PDT	MCF‐7	^[^ ^48^ ^]^
	PZIF67‐AT	CDT	4T1	^[^ ^49^ ^]^
	Copper peroxide nanodots	CDT	U87MG	^[^ ^50^ ^]^
GSH depletion	FA‐Pyrite	CDT	CT26	^[^ ^34^ ^]^
	MMDM (microphage, MCN, DOX, MnO_2_)	CDT/Chemotherapy	4T1	^[^ ^51^ ^]^
	A@P/uLDHs (artemisinin, PEG, MgFe‐LDH)	CDT	HeLa	^[^ ^52^ ^]^
	CCMZ (Cu, CuMoO*_x_*, ZP)	CDT	4T1	^[^ ^53^ ^]^
	JNP Ve (Au‐MnO JNPs, IR1061)	CDT/Radiotherapy	MCF‐7	^[^ ^54^ ^]^
	DMON@Fe^0^/AT	CDT	4T1	^[^ ^55^ ^]^
	CMBP (CuO/MnO*_x_* @BSA, Pt(IV) prodrug)	CDT/Chemotherapy	4T1	^[^ ^56^ ^]^
	Zn*_x_* Mn_1−_ *_x_* S/PDA	CDT/PTT	4T1	^[^ ^57^ ^]^
	PtCu_3_ nanocage	CDT/SDT	4T1	^[^ ^58^ ^]^
*External stimuli*				
Light	DGU:Fe/Dox	CDT/Chemotherapy	4T1	^[^ ^59^ ^]^
	LET‐6 (tPy‐Cy‐Fe, DSPE‐PEG)	CDT/PTT	U87MG	^[^ ^60^ ^]^
	GNR@SiO_2_@MnO_2_	CDT/PTT	U87MG	^[^ ^61^ ^]^
	SnFe_2_O_4_ nanozyme	CDT/PTT/PDT	4T1	^[^ ^62^ ^]^
	MoSe_2_/CoSe_2_@PEG	CDT/PTT	H‐22	^[^ ^63^ ^]^
	HULK (liposome, laccase, MOHQ, FeCe6)	CDT	4T1	^[^ ^64^ ^]^
	Cu‐OCNP/Lap	CDT/PTT	HeLa	^[^ ^65^ ^]^
	AuPd@Fe*_x_* O*_y_*	CDT/PTT	A549	^[^ ^66^ ^]^
	Fe‐doped MoO*_x_* nanowires	CDT/PTT	HeLa	^[^ ^67^ ^]^
	Hollow magnetite nanoclusters	CDT/PTT	HeLa	^[^ ^68^ ^]^
	ICG@Mn/Cu/Zn‐MOF@MnO_2_	CDT/PTT/PDT	U87	^[^ ^69^ ^]^
	Wesselsite (SrCuSi_4_O_10_) nanosheets	CDT/PTT/Starvation therapy	4T1	^[^ ^70^ ^]^
	MoP_2_ nanorods	CDT/PTT	CAL27	^[^ ^71^ ^]^
X‐ray	Cu_2_(OH)PO_4_@PAAS	CDT/Radiotherapy	HeLa	^[^ ^72^ ^]^
	CoFe_2_O_4_ nanoparticles	CDT/Radiotherapy	MCF‐7	^[^ ^73^ ^]^
	TPGS‐Cu_3_BiS_3_	CDT/Radiotherapy	BEL‐7402	^[^ ^74^ ^]^
Ultrasound	Fe_2_P nanorods	CDT	U14	^[^ ^16^ ^]^
	TiO_1+_ *_x_* nanorods	CDT/SDT	4T1	^[^ ^22^ ^]^
	PCF‐a nanozyme (Pd, Cu, Fe)	CDT	4T1	^[^ ^36^ ^]^
	BiFeO_3_ nanocatalyst	CDT	U14	^[^ ^75^ ^]^
	TiO_2_–Fe_3_O_4_@PEG	CDT/SDT	4T1	^[^ ^76^ ^]^
	CoFe_2_O_4_ nanoflowers	CDT/SDT/Immunotherapy	4T1	^[^ ^77^ ^]^
	PLGA‐SPIO&Vc	CDT	MDA‐MB‐231	^[^ ^78^ ^]^
Magnet	HIONCs‐GOD (hollow Fe_3_O_4_, glucose oxidase)	CDT/MHT/Starvation therapy	PC3	^[^ ^79^ ^]^
	FePt‐FeC heterostructure	CDT/NAD^+^ depletion	4T1	^[^ ^80^ ^]^
*Chemical and biological stimuli*				
Gas	FeS@BSA nanocluster	CDT/Gas therapy	Huh7	^[^ ^81^ ^]^
	UCNPs@dMSN‐SNO@CuO_2_‐Ce6‐PEG	CDT/PDT/Gas therapy	U14	^[^ ^82^ ^]^
	FeCO‐MnO_2_@MSN	CDT/Chemodynamic gas therapy	4T1	^[^ ^83^ ^]^
	MnS@BSA	CDT/Gas therapy	4T1	^[^ ^84^ ^]^
Nutrition	GOx‐Fe_3_O_4_‐HMSNs‐PFC/O_2_@C	CDT/Starvation therapy	B16F10	^[^ ^85^ ^]^
	PCN‐224(Cu)‐GOD@MnO_2_	CDT/Starvation therapy	U14	^[^ ^86^ ^]^
	LipoCaO_2_/Fe(OH)_3_‐GOx	CDT/Starvation therapy	MDA‐MB‐231	^[^ ^87^ ^]^
	MCDION‐Se (Nano‐Se, MnCO_3_, Fe_3_O_4_)	CDT/Limotherapy	HeLa	^[^ ^88^ ^]^
Immunization	MDP nanoparticles (PEG‐polyphenol, MnO_2_, DOX, Fe^3+^)	CDT/Chemotherapy/Immunotherapy	B16F10	^[^ ^89^ ^]^
	Cu_2_MoS_4_@GOx	CDT/PTT/PDT/Starvation therapy/Immunotherapy	U14	^[^ ^90^ ^]^
	Cu‐TBP MOF	CDT/PDT/Immunotherapy	B16F10	^[^ ^91^ ^]^
Gene silencing	AIO RNAi nanoparticles	CDT/Gene therapy	PC3	^[^ ^92^ ^]^
	FeCysPW@ZIF‐82@CATDz	CDT/Gene therapy	HeLa	^[^ ^93^ ^]^
*Material design*				
Electron rich nanosystems	BiO_2‐_ *_x_* nanosheets	CDT	4T1	^[^ ^94^ ^]^
	BiFe_0.97_Mn_0.03_O_3_ nanoparticles	CDT	4T1	^[^ ^95^ ^]^
	TOPY‐PEG (2D thermally oxidized pyrite nanosheets)	CDT/PDT/PTT	HepG2	^[^ ^96^ ^]^
	Fe‐MoO_v_ nanozyme	CDT/NADH oxidation	4T1	^[^ ^97^ ^]^
	Fe_3_O_4_/Ag/Bi_2_MoO_6_	CDT/PDT/PTT	4T1	^[^ ^98^ ^]^
Tumor targeting	BDTLAG nanoparticles	CDT/Chemotherapy	MCF‐7	^[^ ^99^ ^]^
	mBMNI (cell membrane, Bi@Bi_2_O_3_@MnO_x_, ICG)	CDT/PTT/PDT	4T1	^[^ ^100^ ^]^
	Pt/GF@Lipo‐TPP	CDT/Chemotherapy	4T1	^[^ ^101^ ^]^
Single atom nanosystems	FeN_3_P‐SAzyme	CDT	HepG2	^[^ ^102^ ^]^
	Pd SAzyme	CDT/PTT	4T1	^[^ ^103^ ^]^
	Mn/PSAE	CDT/PTT	4T1	^[^ ^104^ ^]^
	Cu‐HNCS	CDT	4T1	^[^ ^105^ ^]^

### 4.1 Tumor microenvironment modulation

The modulation of TME properties in favor of Fenton/Fenton‐like reactions can augment chemodynamic cancer therapy efficacy. Lowering pH, increasing H_2_O_2_ concentration, and blocking the activity of antioxidants in TME are major research targets for CDT. Such modulations are often achieved by precise multifunctional naonsystem design.

#### 4.1.1 pH modulation

**FIGURE 2:**
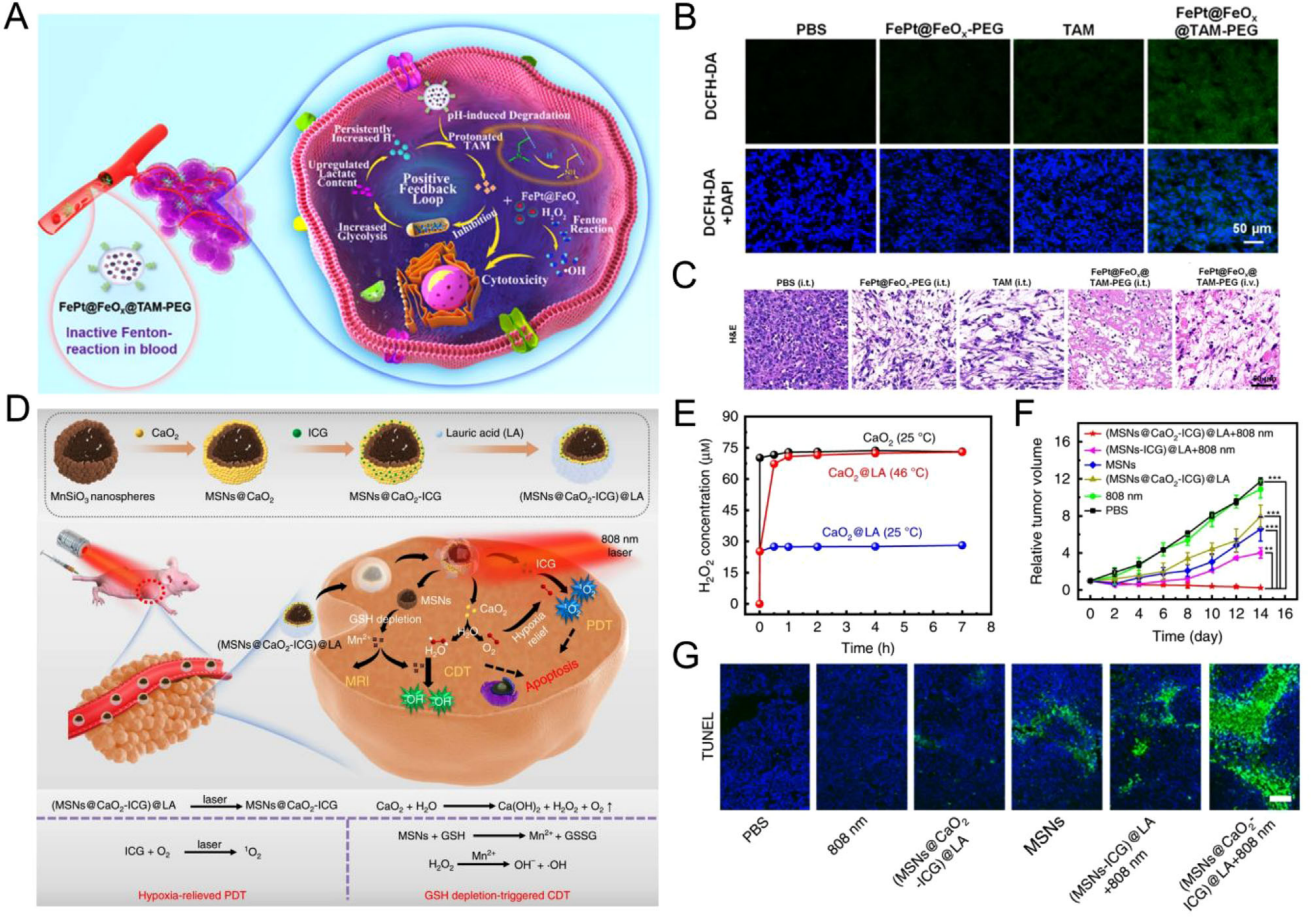
(A) Acidity‐unlocked FePt@FeO*_x_* @TAM‐PEG with positive feedback loop to promote Fenton‐like reactions for self‐boosting tumor specific CDT with minimal side effects. (B) ROS staining images of tumor slices for each group (green color indicates ROS; blue color indicates cell nucleus). (C) Representative images of hematoxylin and eosin (H&E)‐stained tumor slices from each group at the second day post‐irradiation. Reproduced with permission.^[^ ^43^ ^]^ Copyright 2021, Wiley‐VCH. (D) Schematic fabrication process and therapeutic mechanism of thermoresponsive (MSNs@CaO_2_‐ICG)@LA for synergistic CDT/PDT with H_2_O_2_/O_2_ self‐supply and GSH depletion. (E) H_2_O_2_ cumulative release profile ([CaO_2_] = 10 μg mL^−1^). (F) Relative cancer volume changes of MCF‐7 tumor bearing mice after various treatments in 14 days. (G) Terminal deoxynucleotidyl transferase dUTP nick end labeling (TUNEL)‐stained tumor slices collected from different groups at 14 days. Scale bar: 100 μm. Reproduced under the terms of the Creative Commons CC BY license.^[^ ^48^ ^]^ Copyright 2020, The Authors, published by Springer Nature

The Fenton catalysis activity of Fe^2+^ is strongly dependent on solution pH. Fe^2+^ reaches a maximum catalytic activity at around pH 3 through the formation of Fe(OH)_2_. However, the as‐generated Fe^3+^ tends to form inactive hydrous oxyhydroxides at higher pH, diminishing the potential of efficient Fenton reactions.^[^ ^106^ ^]^ The intracellular pH (5–7) of solid tumors poses a significant chemical barrier for efficient Fenton reaction. To overcome this issue, strategies to reduce the intracellular pH of tumors have been proposed. Song and coworkers developed an acidity‐unlocked nanoplatform (FePt@FeO*_x_* @TAM‐PEG) with loaded pH‐responsive tamoxifen (TAM) drug (Figure 2A).^[^ ^43^ ^]^ The release of TAM drug can silence mitochondrial complex I in cancer cells, leading to intracellular H^+^ accumulation through the upregulation of lactate content. The low pH can in turn result in the release of FePt@FeO*_x_* nanocatalyst to generate cytotoxic •OH from H_2_O_2_ through Fenton‐like reaction (Figure 2B). FePt@FeO*_x_* @TAM‐PEG treated cancer cells showed enhanced damage of DNA as compared to the control groups (Figure 2C). The enhanced ROS production was proven to be effective for in vivo therapy. Bu et al. synthesized a nanocomposite (UMP) by coating NaYF_4_:Yb,Tm@NaYF_4_ upconversion nanoparticles (UCNPs) with MIL‐88B MOF along with the loading of photoacids.^[^ ^44^ ^]^ Rapid proton dissociation from photoacids was observed when UMP was irradiated with 980 nm laser, leading to lowered pH in tumor cells. The increment in intracellular H^+^ concentration could accelerate the Fe^2+^ mediated Fenton process for enhanced CDT.

#### 4.1.2 H_2_O_2_ generation

H_2_O_2_ is the main substrate of an efficient Fenton or Fenton‐like reaction. The H_2_O_2_ level in tumor is insufficient for satisfactory CDT efficiency, although the concentration of H_2_O_2_ is in micromolar scale in the solid tumor.^[^ ^107^ ^]^ Thereby, the elevation of the intracellular concentration of H_2_O_2_ is the key. GOx enzyme or artificial GOx‐mimicking nanozymes have been used to produce H_2_O_2_ endogenously by utilizing glucose and O_2_.^[^ ^108^ ^]^ Huang and coworkers opted for a biomineralization strategy to synthesize copper‐doped calcium phosphate (CuCaP) nanoparticles with PEG‐modified GOx as a template.^[^ ^46^ ^]^ After loading DOX, the obtained PGC‐DOX nanocomposite could show acidic TME responsive degradability to release the cargos of Cu^2+^, GOx, and DOX. GOx could effectively catalyze glucose to generate H_2_O_2_, which in turn could accelerate Cu^+^‐mediated Fenton‐like reaction to show enhanced CDT along with chemotherapeutic capacity of DOX.

Additionally, endogenous H_2_O_2_ elevation by chemotherapeutic drugs and SOD‐mimicking enzymes have been exploited. Qu and coworkers constructed a ZIF‐67 based nanozyme (PZIF67‐AT) acting as a H_2_O_2_ homeostasis disruptor.^[^ ^49^ ^]^ The nanozyme could exhibit SOD‐mimicking property to convert O_2_^•−^ to H_2_O_2_, which was utilized in a Fenton‐like reaction to enhance CDT efficacy. Moreover, Wu et al. prepared the Den‐DOX‐tannic acid‐Fe^3+^ nanocomplex by mixing the metal‐phenolic network formed by tannic acid and Fe^3+^ with the DOX loaded dendrimer (Den) for killing cancer cells via an apoptosis/ferroptosis hybrid pathway.^[^ ^45^ ^]^ The improved ROS level triggered by DOX induced apoptosis could sensitize cancer cells to the Fenton reaction induced ferroptosis, thus enhancing the efficacy of CDT. Alternatively, the enhancement of the intracellular H_2_O_2_ concentration could be performed by incorporating H_2_O_2_ precursor, such as, peroxide, in the nanocomposite. Dong et al. fabricated a thermoresponsive nanosystem, namely (MSNs@CaO_2_‐ICG)@LA, comprising of lauric acid (LA) coated and calcium peroxide (CaO_2_) and indocyanine green (ICG) decorated manganese silicate nanoparticles (MSNs) (Figure 2D).^[^ ^48^ ^]^ The photothermal effect could melt thermoresponsive LA layer to expose CaO_2_, which could dissociate into H_2_O_2_ and O_2_ in the presence of water. The generated H_2_O_2_ could enhance the Mn^2+^‐mediated CDT efficacy. The concentration of H_2_O_2_ could be increased up to 70 μM at 46°C, whereas remaining below 30 μM at 25°C (Figure 2E). Owing to the efficient ROS production by the combination of enhanced PDT and CDT, (MSNs@CaO_2_‐ICG)@LA exhibited substantial regression of tumor in vivo (Figure 2F,G).

#### 4.1.3 Glutathione depletion

Regular metabolism in cells is capable of self‐protection by converting the produced ROS into O_2_ or H_2_O via antioxidant mechanism. Such defense systems are mainly comprised of enzymes (GSH‐Px, superoxide dismutase, and catalase), and reducing agents (cysteine, vitamin C, and GSH).^[^ ^107^ ^]^ In cancer tissues, the antioxidant substances are highly overexpressed to counterattack the high level of ROS, endowing tumor cells with the resistance against ROS based therapy. Thereby, the downregulation of such antioxidants, like GSH, is of high impact toward the overall ROS generation for tumor therapy. Several studies have been persuaded to construct nanosystems with GSH depletion property in synergy to Fenton or Fenton‐like reactions to augment the CDT efficiency.^[^ ^109^ ^]^ One of the strategies is to inhibit the intracellular production of GSH by γ‐glutamylcysteine synthetase inhibitor. Liu et al. used L‐buthio‐nine sulfoximine as such inhibitor along with ultrasmall gallic acid‐ferrous (GA─Fe(II)) complex as Fenton reactor inside a liposome for GSH‐depletion enhanced CDT.^[^ ^110^ ^]^ Chen et al. reported that inorganic MnO_2_ had GSH‐depletion abilities and thereby could enhance the CDT performance through Mn^2+^‐mediated OH generation and synergistic disruption of endogenous antioxidant defense mechanism.^[^ ^111^ ^]^ Additionally, nanoparticles possessing disulfide (S─S) or diselenide bond (Se─Se) are useful in depleting GSH via competitive reactions. Bu and coworkers reported a biodegradable nanocarrier (DMON@Fe^0^/AT) by co‐loading iron (Fe^0^) dots and a catalase inhibitor (3‐amino‐1,2,4triazole (AT)) inside S─S bond‐rich dendritic mesoporous organic silica nanoparticles (DMON).^[^ ^55^ ^]^ In mild acidic TME, Fe^2+^ is released to participate in CDT, while DMON persistently depletes endogenous GSH to elevate the oxidative damage.

**FIGURE 3:**
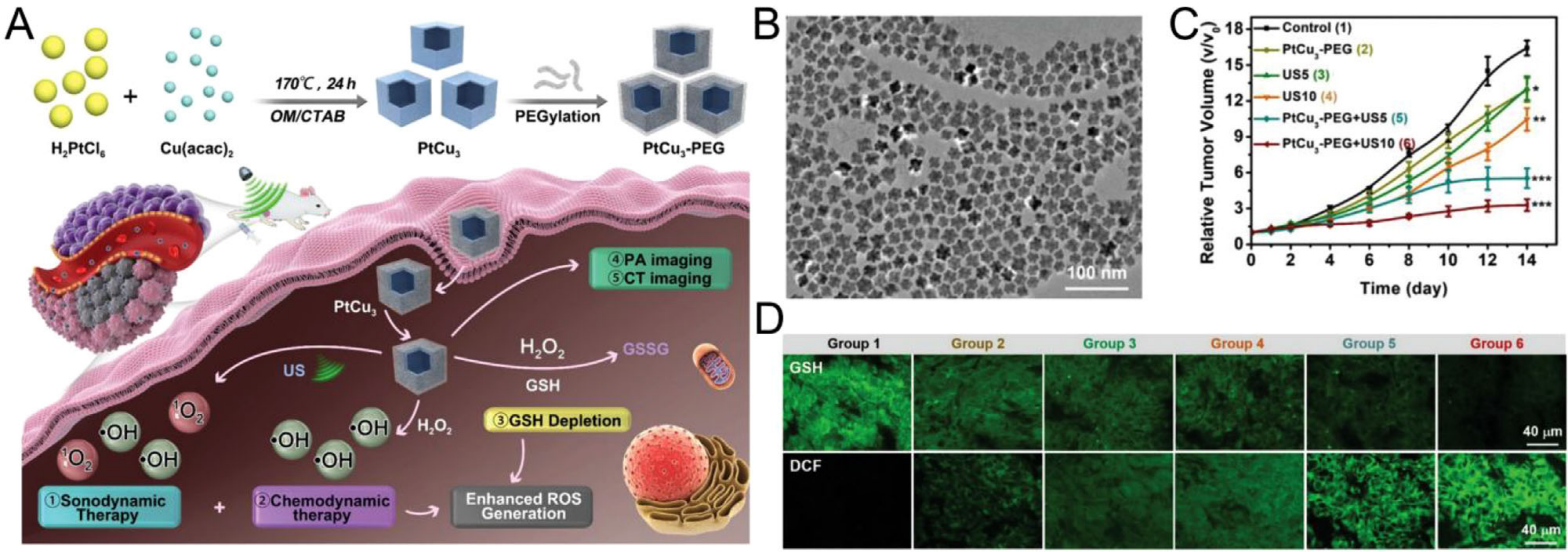
(A) Schematic illustration of photoacoustic (PA)/computed tomography (CT) dual‐modal imaging guided CDT and GSH depletion enhanced SDT by PtCu_3_ nanocages for cancer therapy. (B) TEM images of PtCu_3_ nanocages. (C) Tumor growth curves of mice receiving 1) PBS, 2) PtCu_3_‐PEG, 3) ultrasound irradiation for 5 min, 4) ultrasound irradiation for 10 min, 5) PtCu_3_‐PEG plus ultrasound irradiation for 5 min, and 6) PtCu_3_‐PEG plus ultrasound irradiation for 10 min. (D) GSH and ROS staining of tumors after three rounds of treatments. Reproduced with permission.^[^ ^58^ ^]^ Copyright 2020, Wiley‐VCH

The reduction from Cu^2+^ to Cu^+^ is possible by GSH in TME owing to the low redox potential (∼ 0.16 V) of Cu^2+^/Cu^+^ redox pair, followed by the depletion of GSH concentration. Yang et al. synthesized PtCu_3_ nanocages (PtCu_3_‐PEG) to mimic both horseradish peroxidase (HRP) and GSH‐Px for enhanced CDT (Figure 3A).^[^ ^58^ ^]^ PtCu_3_ nanocages exhibited uniform size and morphology distribution and could act as an HRP‐like nanozyme to decompose H_2_O_2_ into •OH (Figure 3B). Along with ultrasound mediated ROS generation, the GSH‐Px‐mimicking property allowed the nanocages to deplete GSH by the oxidation of H_2_O_2_. Upon ultrasound irradiation, PtCu_3_ nanocages could significantly suppress tumor volumes in vivo (Figure 3C). GSH and ROS staining of the treated tissue sections revealed a proportional relationship between ROS generation and GSH depletion, further confirming the enhancement of oxidative damage through GSH depletion (Figure 3D). Similarly, dual enzyme‐mimicking pyrite nanozymes were synthesized by Fan and coworkers.^[^ ^34^ ^]^ The nanozymes exhibited GSHOx‐like activity to deplete intracellular GSH concentration, which in turn complemented the •OH generation ability through POD‐like activity.

### 4.2 External stimuli

The rate limiting steps of Fenton/Fenton‐like reactions are often governed by the surrounding conditions such as pH and temperature. The utilization of external stimuli, for example, light, ultrasound, and magnet, can provide point‐of‐care therapeutic performance along with the enhancement of reaction rate through promoting the mass transfer ratio.

#### 4.2.1 Light

The induction of Fe^3+^/Fe^2+^ redox cycle in Fenton‐like reaction through photoenergy is a common practice to accelerate the disintegration of H_2_O_2_ into free radicals. Under UV or visible light, Fe^3+^ undergoes a photochemical reaction to form Fe^2+^ along with the generation of •OH from H_2_O_2_. Such short wavelength mediated Fenton and Fenton‐like process has been intensively used in water treatment applications.^[^ ^112^ ^]^ The rate limiting step of Fenton reaction is the reduction of Fe^3+^/Fe^2+^ by H_2_O_2_. The application of photoenergy to improve the efficacy of this step is the foremost motivation of the studies. However, photoinduced Fenton reaction is difficult to be utilized in CDT owing to the low tissue penetration depth of UV/vis light. To tackle this issue, Li and coworkers designed a nanolongan structure, possessing an UCNP core covered by gel particles made from 2,3‐dimethylmaleic anhydride and Fe^3+^ cross‐linked polyethylenimine.^[^ ^59^ ^]^ Upon NIR irradiation, the UV emission by UCNPs promoted the reduction of Fe^3+^/Fe^2+^, which in turn facilitated effective ferroptosis‐apoptosis combined antitumor therapy.

**FIGURE 4:**
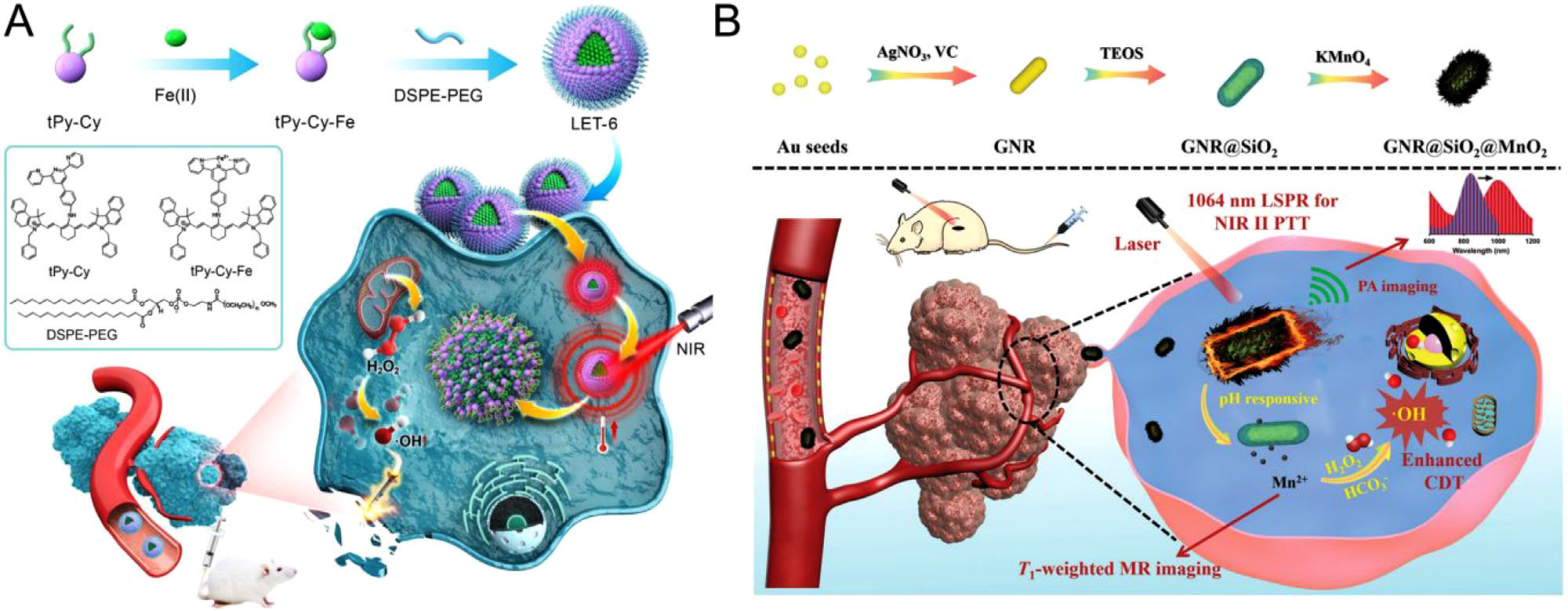
(A) Illustration of an NIR light‐triggered Fe^2+^ delivery agent (denoted as LET‐6) for photothermal enhanced CDT. Reproduced with permission.^[^ ^60^ ^]^ Copyright 2020, Wiley‐VCH. (B) Schematic illustration of the preparation process of GSM nanotheranostic agent for TME‐responsive photoacoustic (PA)/magnetic resonance (MR) dual imaging guided NIR‐II photothermal‐chemodynamic therapy. Reproduced with permission.^[^ ^61^ ^]^ Copyright 2020, Wiley‐VCH

Along with the induction of Fe^3+^/Fe^2+^ redox cycle by photons, the increase in temperature from adjacent photothermal materials can accelerate Fenton or Fenton‐like reaction kinetics. Photothermal systems often absorb wavelengths of NIR‐I window (700–900 nm) and NIR‐II window (1000–1700 nm) for excitation, which can bypass the photon absorption and scattering effect restriction of the tissue components.^[^ ^70^, ^113^ ^]^ Photothermal component in a nanocomposite is utilized to either initiate or synergistically enhance Fenton reaction mediated CDT. Lin et al. reported an NIR light triggered Fe^2+^ delivery agent (LET‐6), comprised of Fe^2+^ chelated 4′‐(amino‐methyl phenyl)‐2,2′:6′,2″‐terpyridine modified cyanine (Figure 4A).^[^ ^60^ ^]^ LET‐6 showed monodispersity with ∼ 50 nm average diameter along with an absorbance peak at ∼ 823 nm, indicating its potential as 808 nm photothermal transducer and photoacoustic contrast agent. The photothermal heating of LET‐6 resulted in thermal expansion of the structure, exposing Fe^2+^ into the TME and inducing augmented CDT through Fenton reaction. The enhanced •OH generation could be detected by the electron spin resonance (ESR) spectra. Additionally, laser irradiated LET‐6 group exhibited significant tumor suppression compared to the control groups. By following a similar idea, Ju and coworkers constructed β‐lapachone loaded metal‐organic coordinated nanoparticles comprised of Cu^2+^ as the node, 1,4,5,8‐tetrahydroxyanthraquinone and banoxantrone dihydrochloride as the organic ligands, and folic acid functionalized PEG as the stabilizing ligand.^[^ ^65^ ^]^ Upon 1064 nm laser mediated photothermal heating and GSH reduction, the released β‐lapachone could trigger an intracellular cyclic reaction to generate abundant H_2_O_2_ for further acceleration of Cu^+^‐mediated Fenton‐like reaction, thus effectively enhancing the CDT efficacy. Recently, Yu and coworkers fabricated molybdenum diphosphide (MoP_2_) nanorods for mild PTT enhanced CDT of oral cancer.^[^ ^71^ ^]^

Moreover, the photothermal effect is often used in synergy with the Fenton reaction to augment CDT. Huang and coworkers constructed a nanocomposite GSM (GNR@SiO_2_@MnO_2_) comprised of gold nanorods (GNR) with silica dioxide (SiO_2_) and manganese dioxide (MnO_2_) coating through a plasmonic modulation strategy (Figure 4B).^[^ ^61^ ^]^ The MnO_2_ layer could be degraded into Mn^2+^ ion upon exposure to endogenous acidity and the released Mn^2+^ ion participated in Fenton‐like reaction for CDT. NIR‐II laser mediated PTT from GSM was utilized to ablate cancer cells in vitro, and the photothermal heating augmented the Fenton‐like reaction. The combinational therapy could suppress tumor volume in vivo, showing the highest apoptotic signaling as compared to the control groups. For another work, Yin et al. fabricated a hollow Mn/Cu/Zn‐MOF with ICG loading and MnO_2_ coating for fluorescence and photothermal imaging guided multimodal therapy (PTT/PDT/CDT). The 808 nm laser mediated photothermal effect could induce local hyperthermia for accelerated •OH generation to enhance CDT. Additionally, CDT can often be used as a light induced therapy in synergism with PDT, and multiple ROS generation induces enhanced oxidative stress in TME.^[^ ^114^ ^]^

#### 4.2.2 X‐ray

**FIGURE 5:**
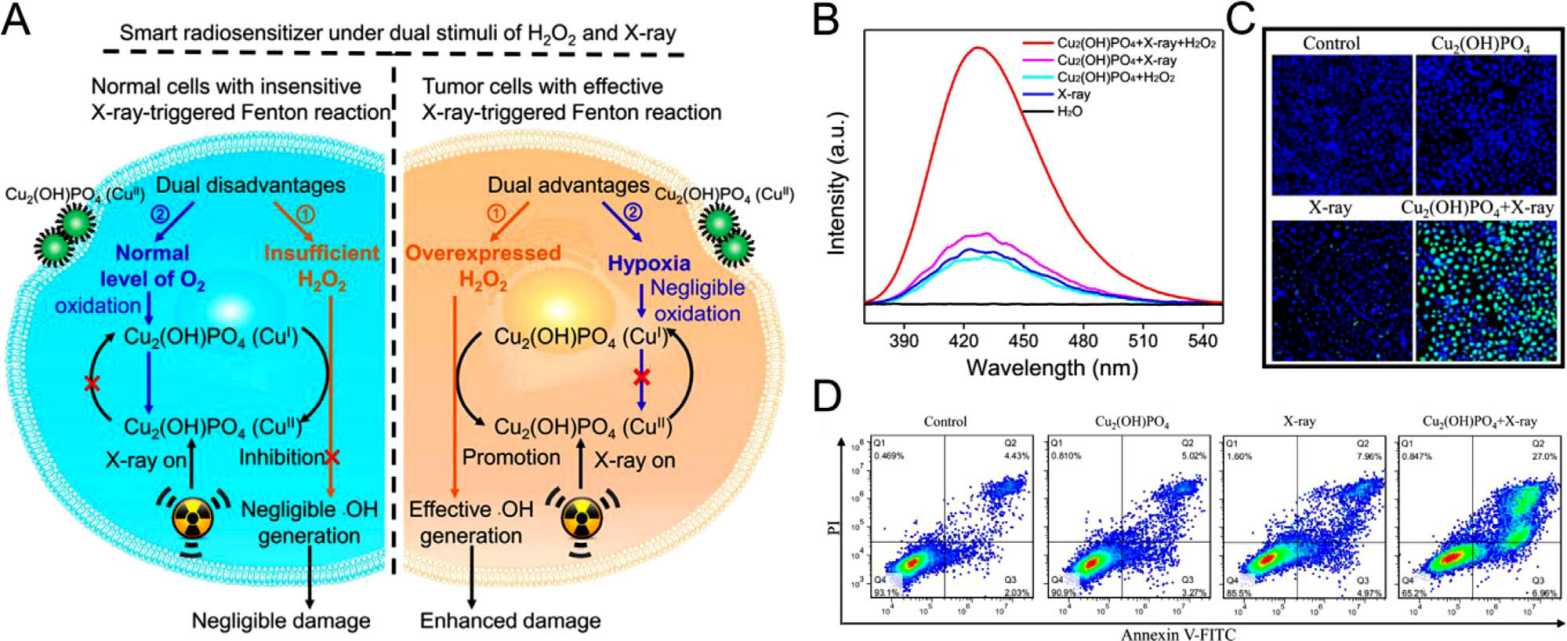
(A) Mechanism of Cu_2_(OH)PO_4_ nanocrystals as a smart radiosensitizer for enhanced radiotherapeutics through X‐ray‐triggered Fenton‐like reaction. (B) Fluorescence spectra of terephthalic acid assay for the detection of •OH generated by Cu_2_(OH)PO_4_. (C) Images of HeLa cells stained with Hoechst 33342 (blue) and DCFH‐DA (green) under different treatments. (D) Flow cytometric analysis of HeLa cells with different treatments. Reproduced with permission.^[^ ^72^ ^]^ Copyright 2019, American Chemical Society

Although researchers have made significant progress for using light as the external stimulus to augment CDT, the penetration depth of light is still less effective for deep tissue tumor treatment. X‐ray possessing a wavelength range of 0.001–10 nm can produce a dose to deeply seated tumors. As a mild dose of X‐ray can be tumor specific by sparing the adjacent healthy tissues, researchers are interested in utilizing X‐ray as a viable physical excitation source. Studies revealed that mild X‐ray dosage can activate the nanomaterial surface to elevate Fenton catalysis efficacy. Zhao and coworkers constructed a smart radiosensitizer based on Cu_2_(OH)PO_4_ nanocrystals, which can generate Fenton active Cu^+^ sites on the nanocrystals under low dose X‐ray irradiation as a result of X‐ray‐induced photoelectron transfer process (Figure 5A).^[^ ^72^ ^]^ Terephthalic acid assay indicated that the nanocrystals could enhance the generation of •OH from H_2_O_2_ under X‐ray induction as compared to the controls, which was further supported by the 2′,7′‐dichlorofluorescin diacetate (DCFH‐DA) assay (Figure 5B,C). Additionally, the nanocrystals under X‐ray irradiation exhibited a substantial number of late apoptotic/necrotic cells in comparison to the other groups, owing to the enhanced CDT (Figure 5D). Zhao and coworkers synthesized Cu_3_BiS_3_ nanocrystals functionalized with amphiphilic D‐α‐tocopherol polyethylene glycol 1000 succinate (TPGS‐Cu_3_BiS_3_) for both NIR‐II light and X‐ray mediated combinational radiotherapy and PTT, along with enhanced CDT.^[^ ^74^ ^]^

#### 4.2.3 Ultrasound

**FIGURE 6:**
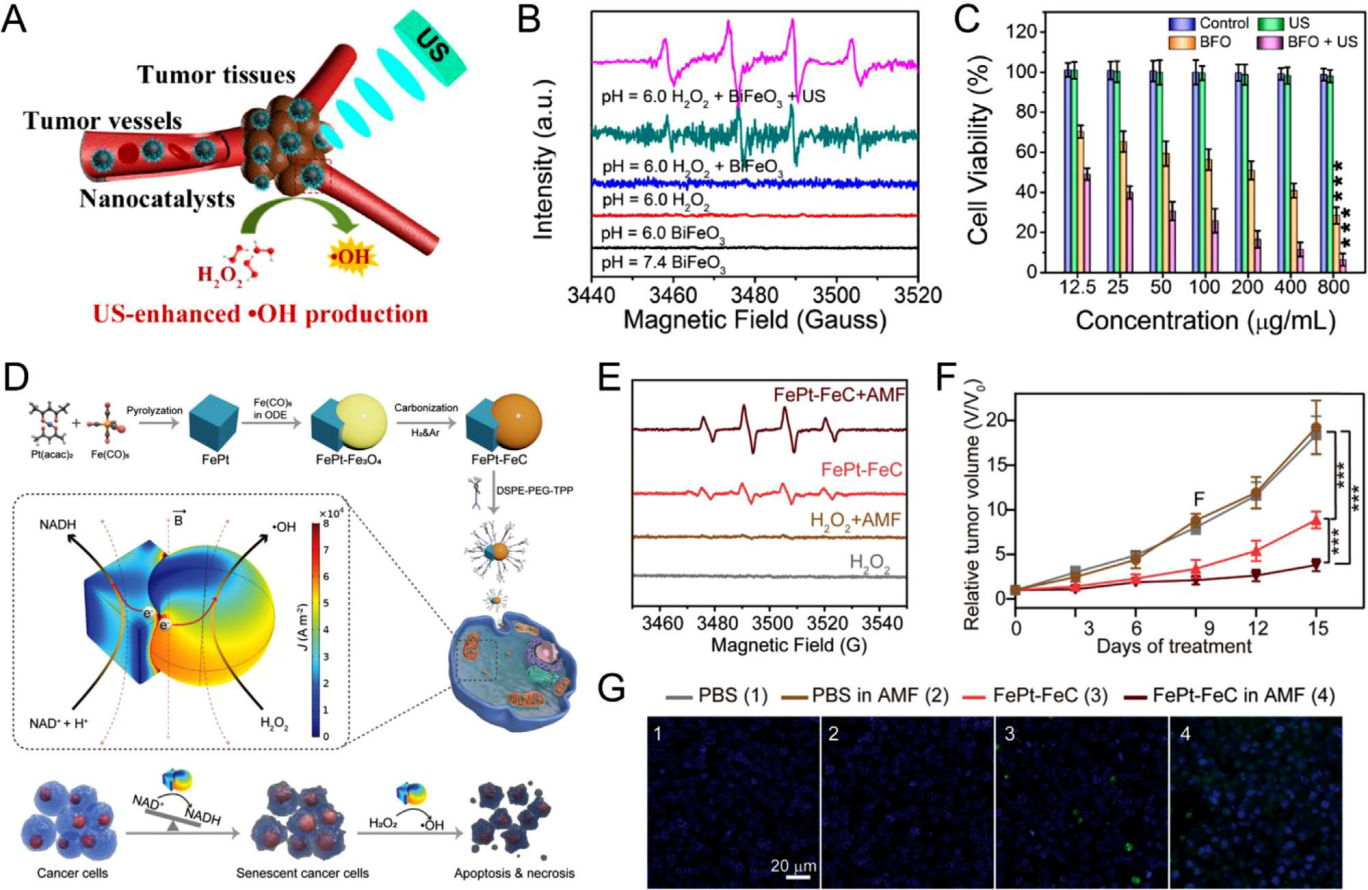
(A) Schematic illustration of ultrasound (US)‐enhanced Fenton reaction in tumor cells. (B) ESR spectra recorded at stated measurement conditions. (C) Cell viability of HeLa cells incubated with BFO with different treatments. Reproduced with permission.^[^ ^75^ ^]^ Copyright 2020, American Chemical Society. (D) Schematic illustration of the three‐step synthetic procedure and the magneto‐electrocatalytic therapeutic strategy of FePt‐FeC heterostructure. (E) X‐band ESR spectra with 5,5‐dimethyl‐1‐pyrroline *N*‐oxide as a spin trap to detect the radical species generated in FePt‐FeC catalyzed Fenton‐like reactions in the presence or absence of AMF. (F) Relative tumor volume growth curves of the 4T1‐tumor bearing mice with different treatments. (G) TUNEL staining images of tumor tissues from the mice after different treatments. Reproduced with permission.^[^ ^80^ ^]^ Copyright 2021, Wiley‐VCH

Ultrasound could generate noninvasive sound waves (∼ 20 kHz), which has been in extensive clinical use for diagnosis and treatment. When travelling through liquid, ultrasound creates concentrated shock waves to produce cavitation bubbles and subsequent intense local vibration.^[^ ^115^ ^]^ The mass diffusion resistance of the substrates can be diminished by ultrasound owing to the acoustic cavitation effect, augmenting the Fenton and Fenton‐like catalytic reaction rates substantially. For instance, Yang and coworkers constructed one‐dimensional ferrous phosphide nanorods for both photothermal and ultrasound assisted CDT.^[^ ^16^ ^]^ Upon exposure to low intensity ultrasound, the conversion rate of Fe^3+^ to Fe^2+^ was accelerated for enhanced CDT, resulting in the tumor ablation in vivo. Cheng and coworkers synthesized ultrafine titanium monoxide (TiO_1+_ *_x_*) nanorods to improve sono‐sensitization and Fenton‐like catalytic activity for ROS therapy of cancer.^[^ ^22^ ^]^ Recently, we reported a one‐pot synthesis of bismuth ferrite nanocatalysts (BFO) for ultrasound‐augmented CDT against malignant tumors (Figure 6A).^[^ ^75^ ^]^ Under ultrasound application, the cavitation effect could enhance the generation of •OH from H_2_O_2_ at TME mimicking conditions (Figure 6B). BFO exhibited significant killing effect to cancer cells under ultrasound in vitro as compared to the control groups (Figure 6C). Additionally, the introduction of low intensity focused ultrasound (LIFU) system could potentially minimize normal tissue damage by focusing on the tumor region through the minimum energy attenuation. Li and coworkers reported a magnetic nanoreactor (PLGA‐SPIO&Vc) comprising of vitamin C and superparamagnetic iron oxide for LIFU‐accelerated Fenton reaction with high tumor killing efficacy.^[^ ^78^ ^]^

#### 4.2.4 Magnet

Magnetic hyperthermia therapy (MHT) is an emerging cancer treatment modality having high tissue penetration and contactless treatment mode. Tumor accumulated magnetic nanoparticles can generate localized heating effect in the presence of an external alternate magnetic field (AMF) without adversely affecting the surrounding normal tissues. The synergy of MHT with CDT could result in effective damage to tumor tissues. Lin et al. constructed a magnetic nanosystem by loading GOx into hollow iron oxide nanocatalysts (HIONCs).^[^ ^79^ ^]^ GOx can stimulate H_2_O_2_ generation through the starvation therapy, followed by the elevation of TME acidity. The Fe^2+^ ion in HIONCs could induce the generation of cytotoxic •OH from the elevated H_2_O_2_ level by Fenton reaction, which was further accelerated owing to MHT‐related temperature rise. Additionally, AMF could simulate magnetically electronic catalysis through the induction of eddy currents in magnetic nanostructures. Bu and coworkers reported a Janus cubic‐sphere FePt‐FeC magnetic heterostructure as a nanoscale catalyst (Figure 6D).^[^ ^80^ ^]^ Under mild AMF, the electron transfer proceeded from FeC to FePt in the heterostructure, leading to the electron density modulation and increased intracellular NAD^+^ reduction efficiency. The applied AMF could further accelerate the Fenton reaction to generate •OH radical (Figure 6E). FePt‐FeC heterostructure treated 4T1 tumor could be eradicated under AMF simulation as compared to the control groups (Figure 6F,G).

### 4.3 Chemical and biological stimuli

Use of external stimuli often focuses on the instantaneous acceleration of the Fenton reaction. Chemical and biological stimuli, such as gas molecules, immune adjuvants, gene silencing, and nutritional components, can be integrated to enhance the postreaction process stimulated by CDT to augment the whole reaction efficacy.

#### 4.3.1 Gas

Gaseous signaling molecules in living systems regulate many physiological and pathological processes. For example, nitric oxide (NO) can be used to sensitize chemicals and radiation, carbon monoxide (CO) can activate caspase through the dysfunction of mitochondria, and hydrogen sulfide (H_2_S) can enhance blood flow in tumor region by reducing blood vessel tension.^[^ ^116^ ^]^ For this aspect, Cai and coworkers synthesized amorphous ferrous sulfide‐embedded bovine serum albumin (FeS@BSA) nanoclusters for H_2_S‐amplified ROS therapy of Huh7 cancer.^[^ ^81^ ^]^ The released H_2_S gas, in response to acidic TME, suppresses the activity of catalase in cancer cells, resulting in enhanced accumulation of H_2_O_2_ and subsequent Fenton reaction by Fe^2+^. NO in cancer cells can rapidly react with ROS to form reactive nitrogen species (RNS), which is more potent than the individual components. Yang et al. fabricated a multifunctional nanocomposite (UMNOCC‐PEG) for acidic TME responsive Cu^2+^, H_2_O_2_, chlorin e6, and NO release, which can enhance CDT and PDT and subsequently generate cytotoxic RNS in the presence of light.^[^ ^82^ ^]^ Moreover, He and coworkers efficiently encapsulated iron carbonyl (FeCO) and MnO_2_ nanoparticles in mesoporous silica nanoparticles for endogenous acidity triggered sequential release of ROS and CO gas for synergistic CDT and chemodynamic gas therapy.^[^ ^83^ ^]^

#### 4.3.2 Nutrition

**FIGURE 7:**
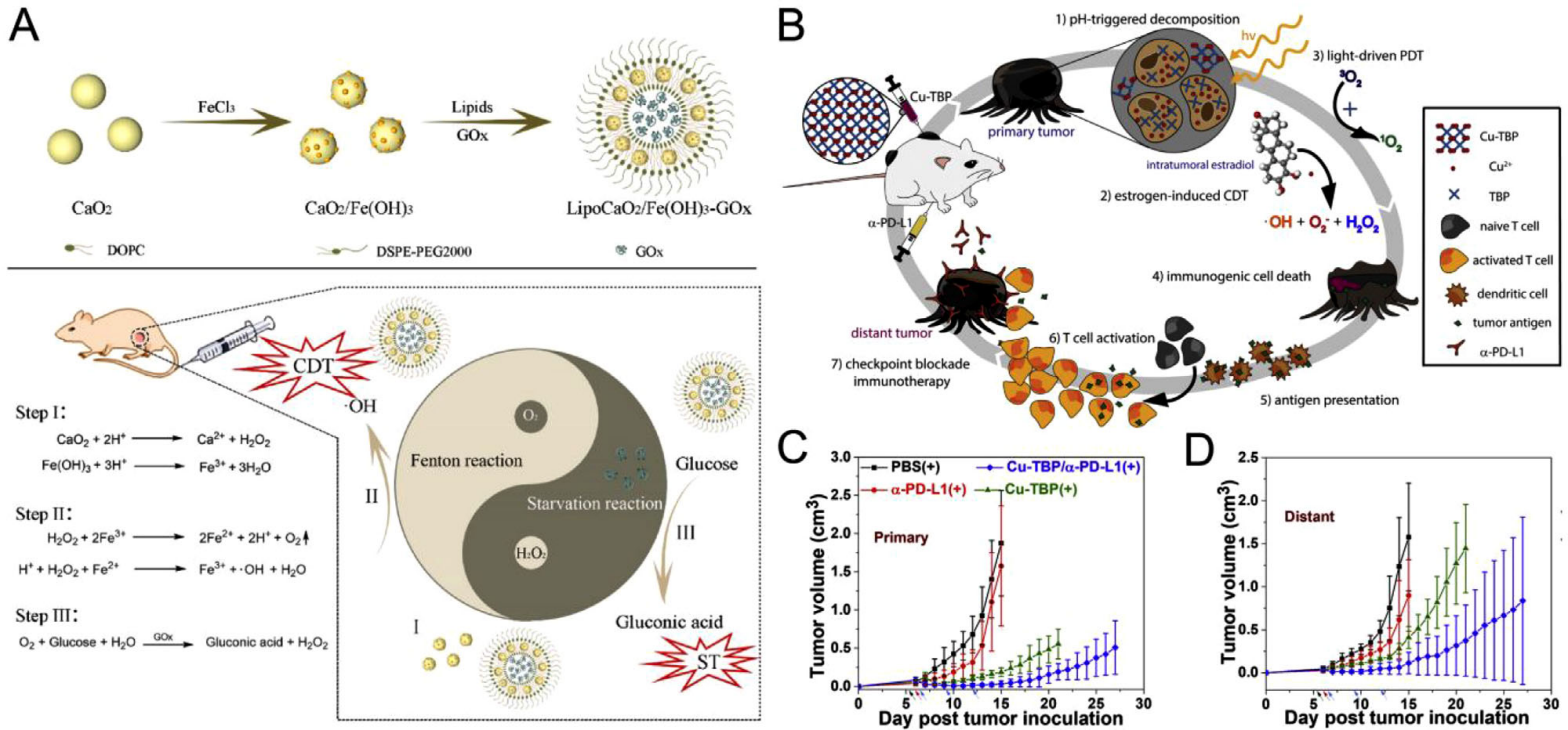
(A) Synthetic procedure of LipoCaO_2_/Fe(OH)_3_‐GOx and illustration of cyclic Fenton/starvation reactions with Tai Ji diagram. Reproduced with permission.^[^ ^87^ ^]^ Copyright 2021, Elsevier. (B) Synergy of checkpoint blockade immunotherapy and nMOF‐mediated radical therapy triggered by both hormone and light stimulation. (C,D) Averaged (C) primary and (D) distant tumor growth curves of B16F10 bilateral tumor‐bearing mice treated with PBS(+), α‐PD‐L1(+), Cu‐TBP(+), or Cu‐TBP(+) plus α‐PD‐L1. Reproduced with permission.^[^ ^91^ ^]^ Copyright 2019, Elsevier

Distinct from the respiratory metabolism of the normal cells, tumor cells rely on the anerobic glycolysis (Warburg effect) for energy production.^[^ ^117^ ^]^ Glucose acts as a key nutritional support for the proliferation and growth of tumor. Thereby, impeding the path of glucose at the source can precipitously consume the nutritional supplement at the tumor region, making it vulnerable to additional therapeutic attack. GOx is identified as a crucial enzyme for tumor starvation therapy, where GOx catalyzes the glucose decomposition in TME to produce H_2_O_2_ that in turn benefits CDT.^[^ ^108^ ^]^ Recently, Xiang and coworkers prepared a nanocomposite (LipoCaO_2_/Fe(OH)_3_‐GOx) by co‐loading GOx and CaO_2_/Fe(OH)_3_ in a biocompatible liposome (Figure 7A).^[^ ^87^ ^]^ Acidic TME can decompose CaO_2_/Fe(OH)_3_ nanostructure to generate H_2_O_2_ and Fe^3+^ for triggering the Fenton reaction. The evolved O_2_ as a byproduct can enhance the catalytic efficacy of GOx for the consumption of glucose to produce gluconic acid and H_2_O_2_, contributing to the elevated •OH production in CDT. The integration of such cyclic catalytic reactions is beneficial for the downregulation of hypoxia‐inducible factor‐1α, promoting effective cell death of hypoxic tumors. An obvious reduction in tumor growth was observed in vivo for LipoCaO_2_/Fe(OH)_3_‐GOx nanoparticle‐treated group through starvation enhanced CDT. In another work, Yang and coworkers constructed fusiform‐like nanoparticles (PCN‐224(Cu)‐GOx@MnO_2_) for synergistic starvation therapy and CDT.^[^ ^86^ ^]^ The MnO_2_ layer catalytically transformed H_2_O_2_ to O_2_, which was sequentially utilized for glucose oxidation through starvation therapy. The released Cu^+^ can react with abundant H_2_O_2_ to produce cytotoxic •OH for CDT. Interestingly, Wu et al. constructed a theranostic nanocomposite, that is, nanoselenium (nano‐Se)‐coated manganese carbonate‐deposited iron oxide nanoparticles (MCDION‐Se), to mimic starvation therapy induced CDT without the use of GOx.^[^ ^88^ ^]^ The nano‐Se exhibited enzymatic activity to produce O_2_^•−^ and H_2_O_2_, followed by transforming to cytotoxic •OH by Mn^2+^ via Fenton‐like reaction. Additionally, the nano‐Se and Mn^2+^ inhibited adenosine triphosphate generation, thus promoting tumor starvation and increasing vulnerability of tumor cells toward CDT.

#### 4.3.3 Immunization

Immunotherapy exploits the congenital immune intervention mechanism to amplify antitumor responses to fight against cancer metastasis and subsequent tumor recurrence. Immunization strategies, such as immune cancer vaccines, cytokine therapy, checkpoint blockade therapy, and T cell therapy, have received substantial interest owing to their promising medical potential.^[^ ^118^ ^]^ However, tumor cells can evade immune recognition via T cell signaling disruption, immune intervention, and tolerance induction. Thus, the induction of acute local inflammation through immunostimulatory treatments can augment tumor immunogenicity and enhance the T cell infiltration to elicit tumor immunity. ROS generated by CDT can promptly induce acute inflammation and immune reactions at the tumor region. For example, Lin and coworkers constructed a degradable nMOF (Cu‐TBP) to mediate synergistic hormone‐induced CDT and light induced PDT (Figure 7B).^[^ ^91^ ^]^ The biodegradable Cu‐TBF nMOF released Cu^2+^ ion in TME, which reacted with intratumoral estradiol to produce H_2_O_2_, •OH, and O_2_^•−^ for CDT. The generated ROS could release tumor‐associated antigens through cell apoptosis to induce immunogenicity, as indicated by higher calreticulin expression in vitro for light induced Cu‐TBP group. Moreover, the synergy of PDT, CDT, and antiprogrammed death‐ligand 1 (α‐PD‐L1) could exhibit both primary and distant tumor regression in vivo through abscopal effect (Figure 7C,D). In another work, Dai and coworkers constructed immunogenic cell death (ICD) inducer nanoparticles (MDP) by the self‐assembly of DOX, phenolic MnO_2_ nanoreactor, Fe^3+^, and PEG‐polyphenols through metal phenolic coordination.^[^ ^89^ ^]^ ROS‐dependent cell death via CDT could accelerate the ICD induction and enhance tumor infiltrating T cell population. Such ICD enhancement approach could effectively increase the response of tumor to PD‐1 checkpoint blockade immunotherapy.

#### 4.3.4 Gene silencing

**FIGURE 8:**
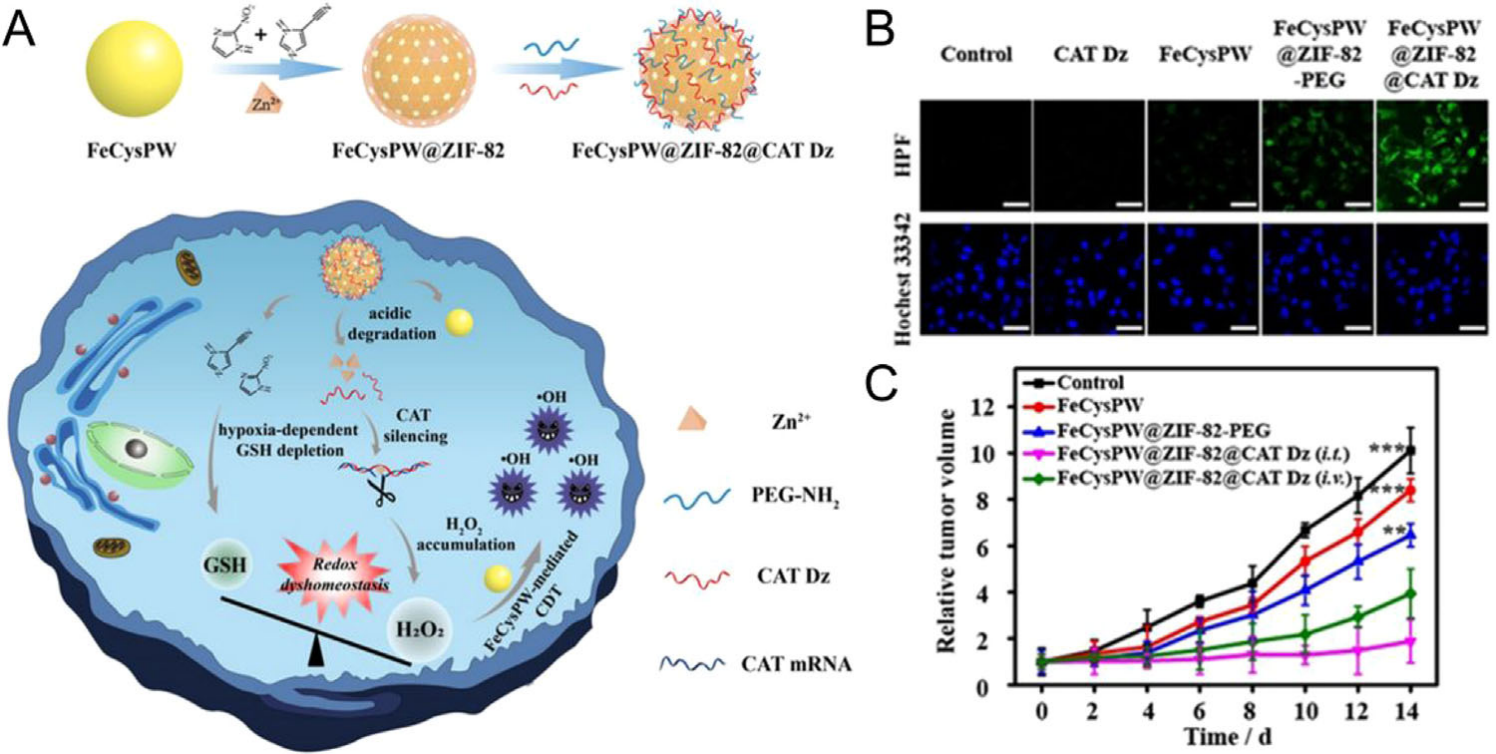
(A) Schematic illustration for the design and multiple functions of FeCysPW@ZIF‐82@CATDz. (B) Intracellular •OH evaluation. Cellular fluorescence images of HPF‐stained HeLa cells treated with PBS, CATDz, FeCysPW, FeCysPW@ZIF‐82‐PEG, and FeCysPW@ZIF‐82@CATDz (Fe, 50 ppm, CATDz, 108 pmol mL^–1^) under hypoxia. Scale bar = 50 mm. (C) Tumor growth curves of tumor bearing mice treated with PBS, FeCysPW, FeCysPW@ZIF‐82‐PEG, FeCysPW@ZIF‐82@CATDz (intratumorally (i.t.)), and FeCysPW@ZIF‐82@CATDz (intravenously (i.v.)). Reproduced with permission.^[^ ^93^ ^]^ Copyright 2020, Wiley‐VCH

Progress of cancer is often correlated with various gene alterations and disorders. Tumor targeted delivery of nucleic acids can modify such gene conditions to potentiate cancer therapy.^[^ ^119^ ^]^ Several cancer gene therapy approaches with high efficacy and less side effects have been reported over the past few years.^[^ ^120^ ^]^ Gene silencing methods have been utilized to customize TME for the enhancement of Fenton and Fenton‐like reactions. Overexpression of monocarboxylate transporters (MCTs) in cancer cells associates themselves with metastasis, angiogenesis, and tumor recurrence, and maintains the intracellular pH homeostasis. Thereby, MCT‐related gene therapy can modulate the TME pH in favor of Fenton/Fenton‐like reaction for enhanced CDT. Shi and coworkers reported an amorphous iron oxide (AIO) RNAi nanoparticle platform to modulate the glycolysis pathway by silencing MCT4 to induce tumor cell acidosis.^[^ ^92^ ^]^ Blocking of intracellular lactate efflux by MCT4 silencing resulted in enhanced H_2_O_2_ production in addition to tumor acidosis, combination of which could amplify intracellular iron‐mediated Fenton reaction and oxidative damage to tumor cells. Additionally, redox homeostasis is often a key factor for ROS tolerance in hypoxic tumor, and the disruption of the same can potentiate ROS‐mediated tumor therapy. Bu and coworkers reported a redox dyshomeostasis (RDH) strategy to combat hypoxic tumor based on a nanoplatform, namely FeCysPW@ZIF‐82@CATDz (Figure 8A).^[^ ^93^ ^]^ The catalase DNAzyme (CATDz) loaded zeolitic imidazole framework‐82 (ZIF‐82@CATDz) shell could degrade in tumor cells into Zn^2+^ as a cofactor for CATDz induced catalase silencing and electrophilic ligands for GSH depletion under hypoxia, both of which led to intracellular RDH and H_2_O_2_ accumulation. Such accumulated H_2_O_2_ further potentiated ferrous cysteine‐phosphotungstate (FeCysPW) induced •OH generation for hypoxic tumor ablation (Figure 8B). The in vivo tumor growth was significantly inhibited by the treatment of FeCysPW@ZIF‐82@CATDz compared to the control groups owing to the RDH‐enhanced CDT (Figure 8C).

### 4.4 Materials design

In addition to the TME modulation and application of stimuli, materials design to modulate the properties for the enhancement of electron transfer favoring Fenton reaction and CDT is a unique approach. Moreover, tumor targeting property of materials endows them with enhanced accumulation in tumor tissues for augmented •OH generation.

#### 4.4.1 Electron rich nanosystems

**FIGURE 9:**
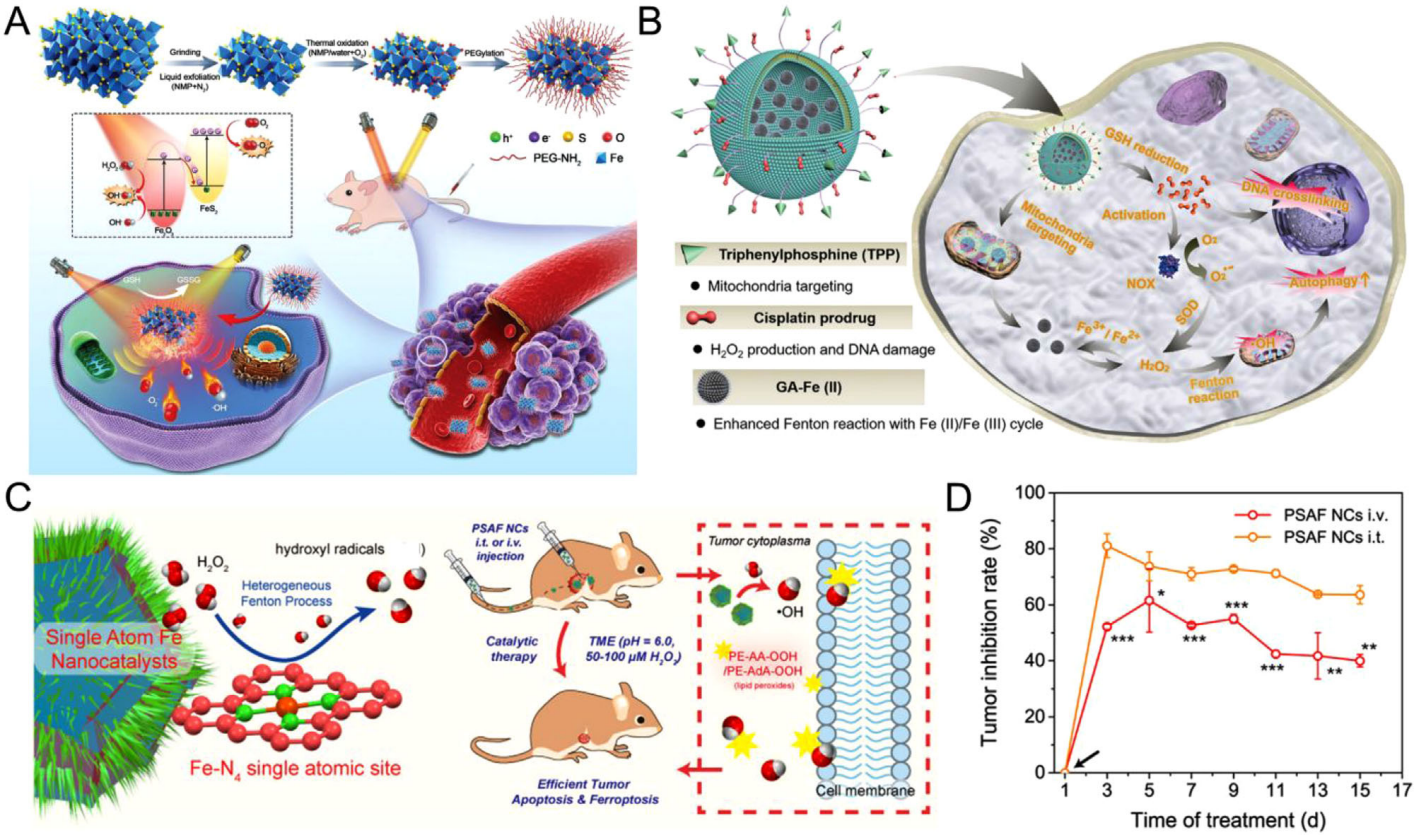
(A) Schematically illustration of preparation and multimodal imaging‐guided cancer theranostic of TOPY‐PEG NSs. Reproduced with permission.^[^ ^96^ ^]^ Copyright 2019, Wiley‐VCH. (B) Schematic diagram of the in vivo chemotherapy‐augmented sequential chemodynamic tumor therapy based on mitochondria‐specific nanocatalysts. Reproduced under the terms of the Creative Commons CC BY license.^[^ ^101^ ^]^ Copyright 2021, The Authors, published by Wiley‐VCH. (C) Schematic illustration of nanocataytic tumor therapy by PSAF. (D) Relative tumor inhibition rates of tumors treated with PSAF intravenously and intratumorally in contrast to control group. Reproduced with permission.^[^ ^121^ ^]^ Copyright 2019, American Chemical Society

The modulation in electron density distribution through materials functionalization can alter the chemical potential of reactive electrons, stimulating substantial influence on the reaction activation energy. The redox cycling of Fe^3+^/Fe^2+^ by H_2_O_2_ is essential for the Fenton catalytic reaction. The introduction of electron‐rich nanomaterials in Fe‐based CDT can facilitate the rate‐determining step to progressively supply Fe^2+^ for H_2_O_2_ decomposition in order to form •OH and reduce the activation energy of Fe^3+^ ion. The charge transfer toward the reaction center Fe atom from non‐reaction‐center atoms is an ideal approach for Fe electron density alteration. Recently, Bu et al. constructed an electron lever by synthesizing 3% Mn^4+^‐doped bismuth ferrite (Bi‐Fe_0.97_Mn_0.03_O_3_) nanocrystals through intra‐crystalline electron lever strategy.^[^ ^95^ ^]^ High valance Mn^4+^ doping can produce unbound free electrons, which can be propelled by the built‐in piezoelectric field into Mn^4+^─O─Fe^3+^ lever inducing electron accumulation on the Fe center. The •OH yield had an 9.21‐fold increment under ultrasound application as compared to the undoped nanocrystals, resulting in significant tumor regression in vitro and in vivo. Moreover, efforts have been made to fabricate Z‐scheme heterojunctions. Upon external stimuli, the electrons in conduction band (CB) of one junction can be transferred to the valance bond (VB) of other junction, creating electron supplementation and subsequent favoring for the ROS generation. Ji and coworkers reported thermally oxidized pyrite nanosheets modified by PEG‐NH_2_ (TOPY‐PEG) with a Z‐scheme heterojunction structure (Figure 9A).^[^ ^96^ ^]^ The photoexcited electrons in CB of Fe_2_O_3_ can be transferred to the VB of FeS_2_, impeding electron–hole pair recombination of FeS_2_ and boosting the redox potentials to generate ROS for CDT and PDT. The generation of •OH and O_2_^•−^ through TOPY‐PEG mediated Fenton reaction and photodynamic reaction was confirmed by the ESR studies. Significant tumor regression was observed in laser irradiated TOPY‐PEG group, further confirming the enhanced ROS production. In another example, Dong et al. fabricated Fe_3_O_4_/Ag/Bi_2_MoO_6_ (FAB) photoactivatable nanozyme for synergistic CDT/PDT/PTT of cancer.^[^ ^98^ ^]^ Doping of Fe_3_O_4_ and Ag nanoparticles endows Bi_2_MoO_6_ nanoparticles with strong NIR‐II absorption and enhanced photocatalytic activities. FAB nanozyme showed multienzyme properties owing to the electron enrichment in the conduction band of Bi_2_MoO_6_ nanoparticles through photoexcitation, migration of excited electrons from Fe_3_O_4_ nanoparticles, and electron injection from Ag nanoparticles.

#### 4.4.2 Tumor targeting

One of the main issues of CDT is the short lifetime and diffusion distance of •OH in TME. Tumor targeting approach can deliver the therapeutic systems directly into the cells, shortening the diffusion distance and directing higher concentration of cytotoxic •OH toward vulnerable biomacromolecules for elevated cell death ratio. Recently, Qiao and coworkers fabricated traceable multistage targeting nanoparticles (BDTLAG) with spatiotemporal CDT efficacy.^[^ ^99^ ^]^ Triphenylphosphine (TPP) and biotin endowed the nanoparticles with mitochondria and tumor targeting ability, respectively. BDTLAG could effectively deliver α‐tocopheryl succinate and lonidamine for targeted CDT, showing the tumor ablation in vivo. Chen et al. also reported a mitochondria‐specific nanocatalyst comprised of cisplatin prodrug and gallic acid‐ferrous (GA‐Fe(II)) for augmented CDT (Figure 9B).^[^ ^101^ ^]^ Additionally, some studies utilized cell membranes to camouflage nanomaterials in order to increase their biocompatibility and homologous targeting. Li and coworkers synthesized 4T1‐tumor cell membrane‐coated bismuth/manganese oxide nanoparticles with high ICG payload (mBMNI).^[^ ^100^ ^]^ Upon specific tumor targeting, the mBMNI could release Mn^2+^ for CDT in response to GSH, along with light induced PTT and PDT.

#### 4.4.3 Single‐atom nanosystems

Heterogeneous catalysis is considered as a surface phenomenon in general. Thus, increasing the surface‐active sites of a catalyst may lead to accelerated catalytic performance. Single‐atom nanoparticles can achieve the maximum catalytic efficiency by utilizing such surface‐active sites in the atomic dimension, showing enhanced specific activity.^[^ ^121^ ^]^ Fenton active atoms like Fe can be used to construct the active sites in the single‐atom catalysts for enhanced CDT. Shi et al. synthesized Fe‐N_4_ based single‐atom nanocatalysts (PSAF) for tumor therapy through local heterogeneous Fenton catalysis (Figure 9C).^[^ ^122^ ^]^ The pyridinic N species in the carbon structure could stabilize the active Fe site and aid to the enhancement of •OH generation via a proton‐mediated homolytic H_2_O_2_ dissociation. PSAF showed high anticancer efficacy through a sustained catalytic process with a tumor regression rate of 63.49% and 40.01% upon intratumoral and intravenous injections, respectively (Figure 9D). Different elements such as Pd,^[^ ^103^ ^]^ Mn,^[^ ^104^ ^]^ and Cu^[^ ^105^ ^]^ have been in use as a replacement for Fe in the single‐atom catalyst for enhanced tumor therapy. Li and coworkers synthesized FeN_3_P‐centered single‐atom nanozyme (FeN_3_P‐SAzyme) to mimic the POD activity of natural enzymes for cancer therapy.^[^ ^102^ ^]^ Computational analysis revealed that the electron donation from the phosphorus atom as well as the less positive charge of the metal Fe center (Fe*^δ^* ^+^) led to the elevated catalytic activity of FeN_3_P‐SAzyme. In vitro cell viability studies indicated that FeN_3_P‐SAzyme had superior •OH generation capability and cell killing efficacy as compared to the control groups. Additionally, FeN_3_P‐SAzyme could obviously inhibit the tumor growth in vivo.

## 5 CONCLUSIONS AND PERSPECTIVE

The unique feature of TME establishes an equilibrium state of elevated ROS and overexpressed antioxidant mechanism in comparison to the normal cells. Additional ROS generation can break this balance and cause oxidative damage toward cancer cells. Over the years, PDT and SDT have been utilized thoroughly to break this ROS equilibrium for eradicating tumor. However, factors like minimal selectivity and possible toxicity of photosensitizers/sonosensitizers, hypoxic TME, and inadequate light penetration depth have limited the use of such therapeutic paradigms. CDT has emerged to be a type of ROS therapy, which utilizes low pH and overexpressed H_2_O_2_ of TME to operate Fenton or Fenton‐like reactions and subsequently releases cytotoxic •OH for oxidative damage to cancer cell protein, DNA, phospholipids, etc. The modification of Fenton or Fenton‐like reactions plays a key part in the progress of CDT. Owing to the complicated nature of TME, conventional Fenton reaction based chemodynamic agents generate insufficient cytotoxic ROS for incomplete eradication of tumor. Higher therapeutic efficacy and lower side effects can be achieved by the modification of the chemodynamic agent design for enhanced Fenton or Fenton‐like reactions.

In this review, we have introduced Fenton and Fenton‐like reactions responsible for cancer therapy and discussed the current research progress of nanosystem design for enhanced CDT. Furthermore, we have outlined the factors for augmenting CDT and how those factors can be incorporated in the design principles of chemodynamic nanosystems. Most of the aspects for enhanced CDT are focused on enhancing the efficiency of Fenton or Fenton‐like reactions through increasing intracellular H_2_O_2_ concentration, decreasing the concentration of reductants, proper design of chemodynamic nanosystems, and applications of physical and chemical stimuli. Although many commendable studies have enriched the field of CDT over the past couples of years, several important matters need to be evaluated and unraveled.

(1) One of the main barriers toward clinical applications of CDT is less systematic research on the long‐term biosafety of chemodynamic nanosystems. Metabolization of the administrated nanosystems is of high importance, and the detailed information about the systemic toxicity and side effects of such nanosystems should be investigated before further applications.
(2) Solid tumor hypoxia is a major issue for cancer therapeutic approach. Being out of reach by the treatment agents, hypoxic tumors are resistant to the treatments and responsible for tumor recurrence and metastasis.^[^ ^123^ ^]^ Although Fenton reactions do not depend on the availability of O_2_, the delivery of chemodynamic agents in hypoxic TME is still a major task. The integration of hypoxia specific treatment modalities with CDT may lead to promising therapeutic outcome.^[^ ^124^ ^]^
(3) Large scale synthesis of nanosystems is of high necessity when considered for clinical applications. In laboratory research settings, only a few milligrams of nanosystems are often synthesized, which need to go through thorough optimization to maintain the morphology for large scale production. Designing straightforward synthetic procedures combined with microfluidic technology may be a way out for overcoming this issue. Computer science and artificial intelligence can be of great prospect for the previsualization of chemodynamic agent structures and morphology as well as predicting the structure‐activity relationship. Such technology can be utilized to design and synthesize personalized CDT nanosystems in large scale and provide personalized treatment solution for different cancer patients. Additionally, computational approaches can be applied toward determining the catalytic efficiency and toxicity of CDT agents like single‐atom nanomaterials.
(4) In depth study of Fenton or Fenton‐like reactions in TME is still required in order to certify such ROS‐based therapy for clinical applications. Generally, studies are proceeded toward in vivo therapy based on the simplistic understanding of in vitro simulated conditions. However, TME is much more complicated owing to the interference of different oxidants, reductants, enzymes, biomacromolecules, etc. Structure‐activity relationship between chemodynamic agents and TME needs to be established for future development of CDT nanosystems.
(5) ROS is overexpressed in tumor tissues as compared to the normal counterparts, and such anomaly makes cancer cells more susceptible toward oxidative damage. Endogenous •OH produced from Fenton or Fenton‐like reactions causes structural damage to cancer cell lipids, DNA, and proteins. Still, there are less trials performed on clinical stage to shed light on issues such as the rate of ROS generation or the extent of oxidative damage in the dynamic TME. Additional attention can be given to the modulation of gene regulation and protein expression to enhance the ROS therapy. The inhibition of glycolysis pathway in cancer cells as well as the induction of G2/M phase arrest may pave innovative ways to sensitize tumor cells to CDT.^[^ ^125^ ^]^ In addition to the elaborate studies on the role of ROS in tumors, the role of ROS in tumor therapy needs thorough investigations in the best interest of future ROS‐based therapeutic strategies.
(6) The design of CDT‐based combinational therapy is another important approach in the cancer therapy, aiming to maximize the CDT efficiency. While CDT is selective for TME and can treat hypoxic tumors, the underwhelmed therapeutic performance still limits the proper utilization. By integrating other therapeutic modalities with CDT, multimodal therapy can be achieved in order to eradicate tumor more efficiently.^[^ ^13^, ^126^ ^]^ The synergy between different therapeutic agents can enhance the potential of individual therapy. However, the aim to construct a nanosystem for multimodal therapy can lead to the complexity in structure, which in turn imposes concerns like non‐biodegradability, non‐biocompatibility, and systemic biotoxicity. The idea for simplifying the composition of chemodynamic agents while achieving multimodal therapeutic outcome needs further research. Additionally, the structures and composition of such nanosystems should be tailored according to the nature of the tumor and the condition of patients for personalized therapy.

Indeed, CDT is a promising cancer therapeutic modality with minimal side effects. In depth investigations of Fenton or Fenton‐like reactions and further modification of the catalytic path would bring along additional possibilities for enhanced CDT. Efforts to answer the unresolved issues of CDT will promote the clinical translation of this approach in coming years. Interdisciplinary research among chemistry, biology, medicine, and materials science can push the boundaries for achieving satisfactory CDT efficacy in cancer treatment, eventually benefiting the patients.

## CONFLICT OF INTEREST

The authors declare no conflict of interest.
